# Who should be prioritized for COVID-19 vaccination in China? A descriptive study

**DOI:** 10.1186/s12916-021-01923-8

**Published:** 2021-02-10

**Authors:** Juan Yang, Wen Zheng, Huilin Shi, Xuemei Yan, Kaige Dong, Qian You, Guangjie Zhong, Hui Gong, Zhiyuan Chen, Mark Jit, Cecile Viboud, Marco Ajelli, Hongjie Yu

**Affiliations:** 1 School of Public Health, Fudan University, Key Laboratory of Public Health Safety, Ministry of Education, Shanghai, China; 2 Centre for Mathematical Modelling of Infectious Diseases, London School of Hygiene and Tropical Medicine, London, UK; 3 Department of Infectious Disease Epidemiology, London School of Hygiene & Tropical Medicine, London, UK; 4 WHO Collaborating Centre for Infectious Disease Epidemiology and Control, School of Public Health, Li Ka Shing Faculty of Medicine, The University of Hong Kong, Special Administrative Region, Hong Kong, China; 5 Division of International Epidemiology and Population Studies, Fogarty International Center, National Institutes of Health, Bethesda, MD USA; 6 Department of Epidemiology and Biostatistics, Indiana University School of Public Health, Bloomington, IN USA; 7 Laboratory for the Modeling of Biological and Socio-technical Systems, Northeastern University, Boston, MA USA; 8 Shanghai Institute of Infectious Disease and Biosecurity, Fudan University, Shanghai, China; 9 Department of infectious diseases, Huashan Hospital, Fudan University, Shanghai, China

**Keywords:** Novel coronavirus disease 2019, Vaccination, Target population, China

## Abstract

**Background:**

All countries are facing decisions about which population groups to prioritize for access to COVID-19 vaccination after the first vaccine products have been licensed, at which time supply shortages are inevitable. Our objective is to define the key target populations, their size, and priority for a COVID-19 vaccination program in the context of China.

**Methods:**

On the basis of utilitarian and egalitarian principles, we define and estimate the size of tiered target population groups for a phased introduction of COVID-19 vaccination, considering evolving goals as vaccine supplies increase, detailed information on the risk of illness and transmission, and past experience with vaccination during the 2009 influenza pandemic. Using publicly available data, we estimated the size of target population groups, and the number of days needed to vaccinate 70% of the target population. Sensitivity analyses considered higher vaccine coverages and scaled up vaccine delivery relative to the 2009 pandemic.

**Results:**

Essential workers, including staff in the healthcare**,** law enforcement, security, nursing homes, social welfare institutes, community services, energy, food and transportation sectors, and overseas workers/students (49.7 million) could be prioritized for vaccination to maintain essential services in the early phase of a vaccination program. Subsequently, older adults, individuals with underlying health conditions and pregnant women (563.6 million) could be targeted for vaccination to reduce the number of individuals with severe COVID-19 outcomes, including hospitalizations, critical care admissions, and deaths. In later stages, the vaccination program could be further extended to target adults without underlying health conditions and children (784.8 million), in order to reduce symptomatic infections and/or to stop virus transmission. Given 10 million doses administered per day, and a two-dose vaccination schedule, it would take 1 week to vaccinate essential workers but likely up to 7 months to vaccinate 70% of the overall population.

**Conclusions:**

The proposed framework is general but could assist Chinese policy-makers in the design of a vaccination program. Additionally, this exercise could be generalized to inform other national and regional strategies for use of COVID-19 vaccines, especially in low- and middle-income countries.

**Supplementary Information:**

The online version contains supplementary material available at 10.1186/s12916-021-01923-8.

## Background

The pandemic is causing unprecedented impact on global health and the economy. In the absence of safe and highly effective vaccines and treatment options, non-pharmaceutical interventions are used to decrease transmission and reduce the burden of coronavirus disease 2019 (COVID-19) but most of these interventions have large economic costs [1]. Effective vaccines against COVID-19 are urgently needed to reduce the significant burden of COVID-19 morbidity and mortality. Globally, there are over 274 vaccine candidates at various stages of development in the research pipeline. Of these, 59 candidates have entered clinical trials [2].

On June 26, 2020, the World Health Organization (WHO) unveiled a plan to deliver 2 billion doses of COVID-19 vaccines, of which 50% will go to low- and middle-income countries, by the end of 2021 [3]. Currently, the projected global production capacity is inadequate to provide COVID-19 vaccines for every human being on the planet, particularly immediately after the first vaccine has been licensed. It is possible that countries and entire regions will have no access to vaccines. For example, COVID-19 cases are rapidly increasing in most African countries [4]. However, none of the COVID-19 vaccine candidates is being developed by an African manufacturer. Even if a vaccine were available, many low-income countries would have to rely on vaccines manufactured abroad. Hence national and multinational vaccine producers will need to allocate a proportion of their production to countries that do not have the financial ability to pre-order vaccine doses that are still to be licensed. Setting priorities for target populations to be vaccinated and optimizing resources within and between countries entails difficult choices. Nonetheless, this is critical for a successful global pandemic vaccination program, and this needs to be addressed urgently. The WHO Strategic Advisory Group of Experts on Immunization (SAGE) Values Framework for The Allocation and Prioritization of COVID-19 Vaccination offers core principles for vaccine distribution [5]. These guidelines need to be further specified and tailored to each county, taking into local contexts including but not limited to the intensity of epidemic, the objectives of pandemic responses, the vaccine supply, and the size of the population eligible for vaccination.

China was the first country to face the COVID-19 pandemic, although only Wuhan, in Hubei Province, was hit by a major wave of infections [6]. Nearly the entire population of mainland China (~ 1.4 billion people) is still susceptible to COVID-19. Recent surges of COVID-19 cases occurred in a growing number of cities such as Beijing, Dalian, Urumchi, and Kashgar, following one or more months without any report of locally acquired infections [7]. There is a risk of a new major wave of COVID-19, especially after the economy and society have re-opened both domestically and abroad.

China has invested substantial resources in vaccines and is one of the main actors in the race to develop a vaccine to help control the COVID-19 pandemic, with resources provided by government, manufacturers, and non-governmental organizations [8]. Eighteen vaccine candidates are being developed in mainland China; five of them are in phase III trials as of November 12, 2020 [9]. New COVID-19 vaccine production facilities recently completed or currently under construction are expected to have the capacity to produce 0.61 billion doses by the end of 2020 and further expanded in 2021 [10]. However, the output is far behind the quantity needed to vaccinate a population of nearly 1.4 billion people in mainland China alone (given a two-dose schedule for all vaccine candidates).

The Joint Prevention and Control Mechanism of the State Council roughly divides the target population for COVID-19 vaccination into three groups, including those with high risks of exposures to the novel severe acute respiratory syndrome coronavirus 2 (SARS-CoV-2), those with high-risks of severe outcomes, and the general population, with priority given to the former two groups [10]. In July 2020, three COVID-19 vaccines were licensed in China for emergency use among individuals at high risk of exposure to SARS-CoV-2, including frontline medical personnel and overseas workers in China. Media reports show that over one million people have been vaccinated as of December 1, 2020 [11, 12]. According to recent surveys [13–15], the (general) Chinese population has a high level of willingness to accept COVID-19 vaccination. Hence, with more vaccines expected to be licensed by the end of 2020/early 2021, there is a need to define the priority target groups for a wide-scale COVID-19 vaccination program. This study aims to define the priority target populations, their size, and priority for a phased introduction of COVID-19 vaccination with evolving goals in mainland China, accounting for risk of severe illness and transmission. This approach is generalizable to inform national and regional strategies for the use of COVID-19 vaccines, especially in low- and middle-income countries.

## Methods

### Goals of the COVID-19 vaccination program

Using the Pandemic Severity Assessment Framework [16], developed by the United States (US) Centers for Disease Control and Prevention to determine pandemic influenza severity, the COVID-19 pandemic can be characterized as having both very high transmissibility and clinical severity [17]. The overarching goal of a vaccination program in the midst of such a pandemic is to vaccinate all persons willing to be vaccinated. However, due to limited supplies, prioritization is warranted. The specific goal of COVID-19 vaccination in China could be determined in a phased approach. In this early phase, the most important objective (*primary goal*) of the vaccination program is to maintain essential services (e.g., healthcare and national security) [18, 19]. The second objective (*secondary goal*) is to reduce the number of individuals with severe outcomes, including hospitalizations, critical care admissions, and deaths [18, 19]. In later stages, the objective of the vaccination program can be further extended to reduce symptomatic infections and/or to stop virus transmission (*tertiary goal*). These goals should be adapted along with the evolving dynamic of the epidemic and an increase of vaccine supplies. We accordingly refer to this approach as a phased universal vaccination program.

### Priority population groups for a COVID-19 vaccination program

**Fig. 1:**
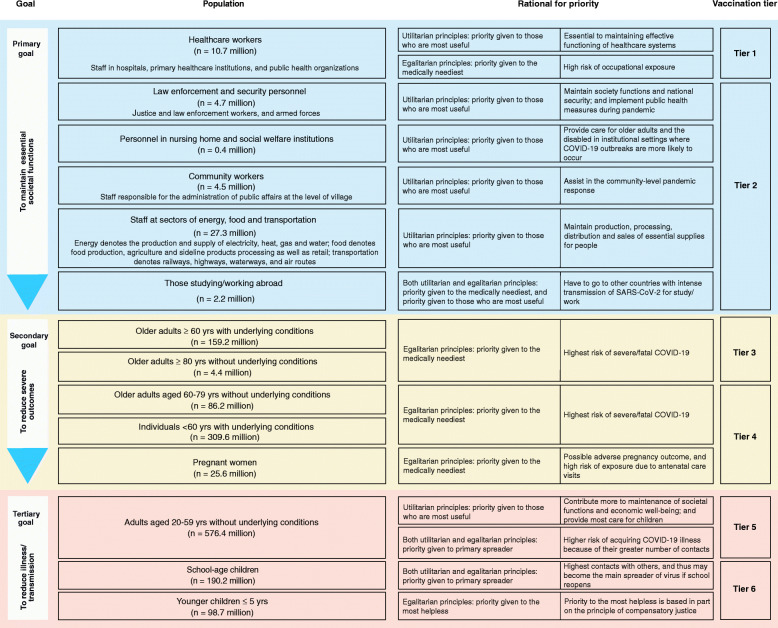
Prioritized segments of the population for a COVID-19 vaccination program as well as estimated population size

In line with the aforementioned goals of a COVID-19 vaccination program, prioritization is based on utilitarian (i.e., maximizing health and economic benefit) and egalitarian (i.e., protecting the worst off) principles. We define population groups to be prioritized by occupation, age, and underlying conditions, taking account (1) the interim framework for COVID-19 vaccine allocation and available guidance on allocating vaccines during an influenza pandemic [e.g. from the WHO, US and the United Kingdom (UK), see summary in Additional file 1: Table S1 [5, 18, 20]], (2) the objectives of and experience gained from the 2009 H1N1 pandemic vaccination program in China [21], (3) specific high-risk groups for severe COVID-19 outcomes and high-risk groups for exposures, and (4) lessons learned from the response to the COVID-19 outbreak in Wuhan such as the role of critical workers in sustaining essential societal functions [1] (Fig. 1). Priority groups include (1) essential workers, including but not limited to healthcare workers (utilitarian principles); (2) high-risk individuals such as those at the highest risk of severe/fatal outcomes (egalitarian principles); (3) individuals who play a key role in transmission (both utilitarian and egalitarian principles) [22]. Generally, within the populations of interest for the primary and tertiary vaccination goals, the target population groups that met ≥ 2 of the aforementioned principles were assigned to a higher tier. For the secondary goal, the target population at higher risk of severe/fatal COVID-19 outcome was assigned to a higher tier. Subsequently, these population groups were categorized into six vaccination tiers in order of decreasing priority. Across priority population groups, vaccines can be allocated and administered according to tier, which means that all groups within a tier have equal priority for vaccination.

#### Essential workers

Individuals who are critical for preserving essential societal functions for public health and safety as well as the well-being of the community during a pandemic include (1) first responders who may have close contact with potential COVID-19 patients in professional settings, including healthcare, public health, and community workers (these include staff in community service agencies, who maintain supply of daily essential needs for people under lockdown, and take routine prevention measures such as fever screening and environmental disinfection); (2) individuals who are essential for maintaining national security, namely individuals working in law enforcement agencies and security personnel (police and military); (3) workers maintaining production and supply of daily essentials, including energy, water, food, and transportation.

Healthcare workers are essential in maintaining an effective healthcare system, not only for COVID-19 but also for other healthcare needs. They often have a high risk of infection due to occupational exposure. For instance, healthcare workers performing endotracheal intubation had a higher risk of SARS-CoV-2 infection than other healthcare workers (odds ratio 4.33, 95% confidence interval 1.16–16.07) since they had higher exposure associated with aerosol-generating procedures [23]. Public health staff also play a crucial role in the COVID-19 response, with responsibilities including, but not limited to, case detection, isolation, tracing, and testing of close contacts, surveillance, and health communication.

An additional category of essential workers includes staff in nursing homes and social welfare institutions, who provide care for older adults and disabled persons in institutional settings, where COVID-19 outbreaks could be devastating [24]. Police are necessary for the society to function and in China are also responsible for implementing a set of public health measures such as tracing of cases and close contacts, and isolation. In addition to maintaining national security, the military also plays a key role in COVID-19 response, as they can provide medical care and support the implementation of prevention and control strategies. Further, community network members are needed to assist in local pandemic response such as mass screening and provide support for vulnerable populations such as seniors, those living alone, and households complying with voluntary quarantine when a household member is ill. The energy (electricity, oil, fuel, and natural gas), water, food, and transportation sectors maintain production, processing, distribution, and sale of essential supplies for the population. These personnel are critical to providing essential goods and services, and thus need to work even during periods of community restrictions, social distancing, or closure orders. Moreover, given the current epidemiological situation in China, characterized by a nearly entirely susceptible population and very limited local transmission, individuals working or studying abroad may have a higher risk of exposure to SARS-CoV-2 compared to domestic residents. As such, this class of individuals has been included in the vaccine emergency programs already implemented in Beijing, Sichuan, and Wuhan and is thus included in Tier 2 in the present study.

Accordingly, we recommend these individuals to be an appropriate first-level priority target group for vaccination. We obtained the population size stratified by occupation from publicly available data, including the China Economic Census Yearbook 2018, the Tabulation the 2010 Population Census of the People’s Republic of China, White Paper on China’s National Defense, Ministry of Education, Ministry of Commerce, and published literature [25–30].

#### High-risk individuals

To meet the secondary goal of the vaccination program, individuals who are at increased risk for severe outcome of COVID-19 could be considered a priority target population for vaccination. We conducted a narrative literature review in PubMed, Embase, Web of Science, medRxiv, and bioRxiv for systematic reviews written in English, to identify the risk factors of severe illness associated with COVID-19. We searched for (“severe” OR “severity” OR “critical” OR “hospitaliz*” OR “ICU” OR “death*” OR “mortality” OR “fatal”) AND (“risk factor*”) AND (“2019-nCov” OR “COVID-19” OR “COVID 19” OR “2019 novel coronavirus” OR “coronavirus disease 2019” OR “SARS-CoV-2” OR “SARS CoV 2” OR “severe acute respiratory syndrome coronavirus 2”).

Clark and colleagues extracted the prevalence of underlying health conditions from the Global Burden of Diseases, Risk Factors, and Injuries Study (GBD), and estimated the number of people with at least one of these conditions in 2019 for 188 countries [31]. Using Clark’s method, we updated the probability of having at least one of these conditions for China to additionally include the prevalence of body mass index ≥ 30, which were identified as risk factors by our review. Then we estimated the age-specific population size of individuals with any of these conditions by multiplying the estimated probability by the United Nations (UN) mid-year population estimates for 2020 for China [32]. The population size of individuals without these conditions was calculated subtracting those with health conditions from the total population.

Pregnant women were additionally included in the list of high-risk groups. We estimated the number of women who are pregnant in 1 year as the sum of all live births, still births, fetal deaths, and abortions in that year. The number of live births was obtained from China Health Statistical Yearbook in 2020 [33]. The number of still births and fetal deaths was estimated as the product of the number of perinatal deaths and the fraction of those deaths which are still births and fetus deaths (68.59%) [34]. We estimated the number of abortions by dividing the number of induced abortions by the proportion of induced abortions (88.54%) [35].

#### Individuals at high risks of symptomatic COVID-19 infections

A second narrative literature review was conducted to assess the risk of symptomatic COVID-19 infection, using the search query, (“2019-nCov” OR “COVID-19” OR “COVID 19” OR “2019 novel coronavirus” OR “coronavirus disease 2019” OR “SARS-CoV-2” OR “SARS CoV 2” OR “severe acute respiratory syndrome coronavirus 2”) AND (“incidence” OR “attack rate” OR “morbidity”) AND (“age profile” OR “age group” OR “age range” OR “age structure” OR “age composition” OR “age spectrum”). Based on the identified risk factors for symptomatic COVID-19 infections, we defined the target populations for vaccination that would help meet the tertiary goal of reducing illness. The populations size was obtained from UN mid-year population estimates for 2020 for China [32], and Ministry of Education of China [36].

### Estimating size of target population of the phased universal vaccination program

First, we estimated the corresponding population size separately for each target population as mentioned above. When a person is included in more than one group, she/he is intended to be vaccinated in the highest tier group in which she/he is included. Accordingly, we then excluded people in more than one risk group to estimate the total population size stratified by goals of vaccination in different phases of the pandemic, and by vaccination tiers.

Assuming vaccine efficacy (VE) around 85–90%, as preliminary analyses of phase 3 clinical trials of several COVID-19 vaccines seem to suggest [37], and considering the basic reproduction number (R_0_) = 2.5, as found in previous studies about China [38], we used the well-known eq. (1 − 1/*R*_0_)/VE to estimate the minimum fraction of population to be immunized to reach herd immunity [39]. The resulting estimate of the vaccination coverage is around 70%, in agreement with previous studies [40]*.* As a sensitive analysis, we considered more conservative estimates of vaccine efficacy (around 65–70%) and thus a vaccination coverage of 90%. Those two scenarios align well with estimates of willingness to be vaccinated against COVID-19 in the Chinese population, namely 72.5–91.3% according to three surveys [13–15]*.* We therefore estimated the days needed to vaccinate 70% of the targeted population in the sequence of tiers given a two-dose vaccination schedule, without accounting for issues in production capacity (see schematic diagram in Additional file 1: Fig. S1).

During the 2009 influenza pandemic, a maximum of 3 million daily doses of pandemic influenza H1N1 (H1N1pdm) vaccines were administered in China [41]. However, the willingness to be vaccinated against COVID-19 is higher than that for the 2009 H1N1 pandemic [13, 42]. Moreover, the vaccine distribution capacity is likely to be improved as well, spurred by the progressive enhancement of the roll-out of Supplementary Immunization Activities in Children in the last decade [43]. As such, we assumed that the capacity of COVID-19 vaccination services could be scaled up to 10 million doses administered per day in the baseline analysis. Sensitivity analyses on the daily doses administered (3 and 20 million) were conducted as well. We also conducted a sensitivity analysis using an uptake rate of 90%.

## Results

Figure 1 illustrates the priority population groups and the corresponding population size estimated without excluding duplicates between groups.

### Essential workers

It is important to stress that the vaccine may be in extremely short supply when first available. To meet the primary goal of vaccination, thus it could be necessary to consider healthcare workers as the top priority (Tier 1 of the vaccination strategy) based on utilitarian and egalitarian principles. Law enforcement and security workers, personnel in nursing home and social welfare institutes, community workers, workers in energy, food, and transportation sectors are included in Tier 2 based on utilitarian principles. Those studying/working abroad are also included in Tier 2 based on utilitarian and egalitarian principles (Fig. 1). We estimated that in mainland China there are 10.7 million healthcare workers, 4.7 million people working in law enforcement agencies and security personnel, 0.4 million personnel in nursing home and social welfare institutes, 4.5 million community workers, 27.3 million workers in the energy, food, and transportation sectors, and 2.2 million persons studying/working abroad.

### High-risk individuals

Over 50 published systematic reviews reported the pooled risk of severe outcome of COVID-19 (Additional file 1: Table S2). These reviews showed that an increased risk of severe outcomes from SARS-CoV-2 infection was observed in individuals with chronic respiratory disease, heart disease, cardio-cerebrovascular disease, hypertension, diabetes, chronic renal diseases, chronic liver disease, cancer, and obesity [44–51] (Additional file 1: Table S2). One systematic review evaluated the disease severity of COVID-19 during pregnancy and found that 21% were severe/critical cases [52]. COVID-19 may cause fetal distress, miscarriage, respiratory distress, and preterm delivery, although evidence for these associations is still inconclusive [53]. Moreover, pregnant women have high frequency of antenatal care visits and thus have a possibly higher exposure to SARS-CoV-2. Although no systematic review found a significantly higher risk of severe outcomes for those with immunodeficiency/immunosuppression, chronic neurological disorders, and sickle cell disorders, we included these categories in our analysis as recommended by the US and UK [47, 54–56].

Age is one of the most important risk factors for severe/fatal COVID-19. Our systematic reviews showed that individuals age ≥ 60 years had about 4-fold higher risk of severe/fatal COVID-19 than younger people (Additional file 1: Table S2). Wu et al. found that the case-fatality risk for those aged ≥ 80 years was 1.7–3.6 times that among those aged 70–79 and 60–69 years [57]. Age and underlying conditions combine to increase the risk [58]. Accordingly, adults ≥ 60 years of age with underlying conditions, and adults ≥ 80 years of age without underlying conditions, who are at the highest risk of severe/fatal COVID-19, were considered in Tier 3, based on egalitarian principles. Compared to these persons, the risk of severe/fatal COVID-19 among older adults aged 60–79 years without underlying conditions and individuals < 60 years of age with underlying conditions was lower. These individuals aged < 60 years with pre-existing medical conditions and pregnant women were included in Tier 4 based on egalitarian principles (Fig. 1).

We estimated that 309.6 million individuals aged < 60 years and 159.2 million individuals aged ≥ 60 years had at least one high-risk medical condition in mainland China. The number of pregnant women was thus estimated at 25.6 million in mainland China (Fig. 1).

### Individuals at high risks of symptomatic COVID-19 infections

Population-based studies demonstrated that the incidence of COVID-19 cases in those aged 20–59 years was similar to that among older adults [6, 59] (Additional file 1: Table S4). Our meta-analysis showed the cumulative incidence was 139–161 per 100,000 persons among those aged 20–59 years, which was comparable to incidence in those aged ≥ 60 years (195 per 100,000 persons) (Additional file 1: Fig. S2). These working-age adults had a higher risk of acquiring COVID-19 symptomatic infection possibly because of their large number of contacts at work and in the community [60]. Additionally, they contribute to maintenance of societal functions and economic well-being; and they generally provide care for children. Given these considerations, individuals aged 20–59 years without underlying conditions (*n* = 576.4 million) were included in Tier 5 based on both utilitarian and egalitarian principles (Fig. 1).

Population-based sero-epidemiological studies also reported lower seroprevalence in children than in adults [61, 62]. Whether this reflects lower susceptibility of children to infection in general, or similar infection rates, but much higher proportions with asymptomatic disease, or rather the effect of school closures, the implemented strict social distancing measures, or a self-protective behavior of the population remains unclear. Modeling studies found conflicting results about the effect of interventions targeted at children on SARS-CoV-2 transmission at the community level [63, 64], suggesting that there is still uncertainty surrounding fundamental epidemiological features of COVID-19 (e.g., children’s infectiousness [65, 66], susceptibility to infection [64, 67], and probability of developing symptoms) [68]. To ensure the continuity of educational activities, and reduce transmission, school-age children (*n* = 190.2 million) are recommended for vaccination in Tier 6 based on both utilitarian and egalitarian principles (Fig. 1).

The incidence of COVID-19 was lower in younger children. However, the severity among young children has not been fully addressed. Verdoni et al. reported an outbreak of a novel severe Kawasaki-like disease in children related to COVID-19 in Italy, which raised concerns about the impact of the pandemic on younger children [69]. Considering such possible post-infectious inflammatory syndrome as Kawasaki-like disease, younger children aged ≤ 5 years (*n* = 98.7 million), which are priority groups for influenza vaccination, are recommended in Tier 6 as well, based on egalitarian principles of prioritizing the most vulnerable individuals (Fig. 1).

### Estimated size of target population of the phased universal vaccination program

**Fig. 2:**
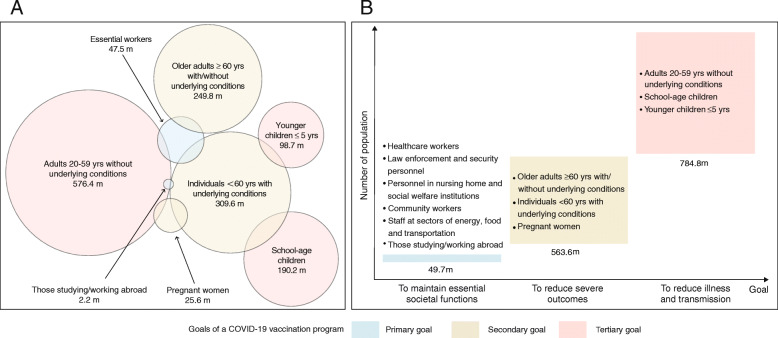
Estimated size of target population for the COVID-19 vaccination program by goal. **a** Overlap of target population groups. **b** Estimated number of targeted individuals excluding the overlaps between groups. Note that m denotes million

**Fig. 3:**
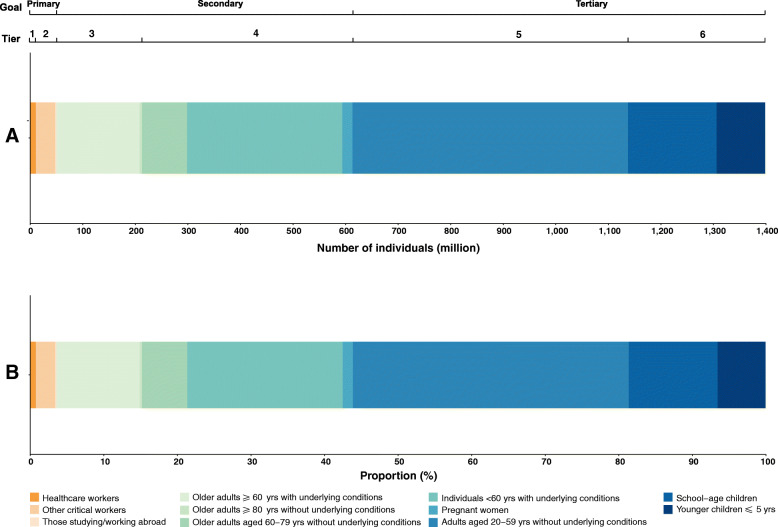
Estimated size of target population for the COVID-19 vaccination program by population group. **a** Number of individuals. **b** Proportion. Note that the overlaps between groups were excluded

**Fig. 4:**
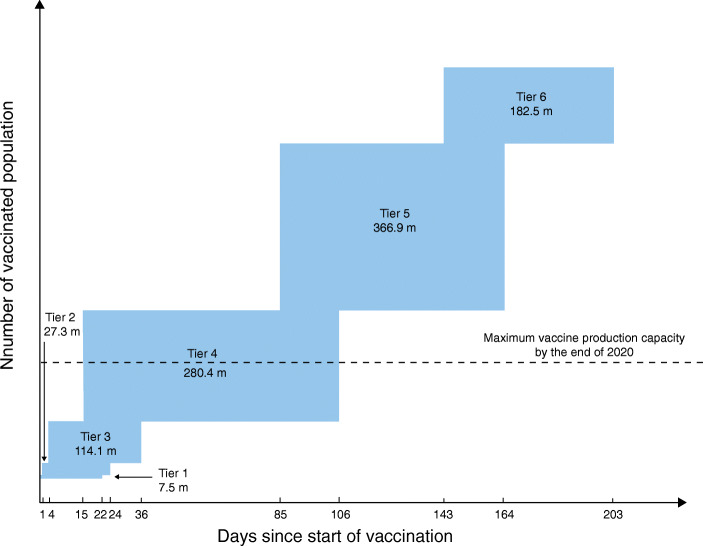
Days needed to vaccinate 70% of the target population, stratified by vaccination tier, under the assumption that 10 million doses are administered per day. Note that values reported within the square (e.g., 182.5 m) denote 70% of the population size in each tier; m denotes million

To maintain essential societal functions, the target population of vaccination was estimated at 49.7 million (Tiers 1 and 2, Fig. 1 and Fig. 2). An additional 563.6 million persons were included in the target population if the goal of vaccination was extended to reduce the number of severe COVID-19 cases (Tiers 3 and 4, Fig. 1 and Fig. 2). Along with the increase of vaccine supply, the remaining 784.8 million persons could be further targeted for vaccination to reduce the total number of COVID-19 symptomatic cases and potentially halt transmission (Tiers 5 and 6, Fig. 1 and Fig. 2). In terms of vaccination tiers (from Tier 1 to Tier 6), a total of 10.7, 39.0, 162.9, 400.6, 524.1, and 260.7 million persons were included in the target population (Figs. 1, 3 and 4).

Given 10 million doses administered per day, and a two-dose vaccination schedule, it will likely take about 7 months to vaccinate 70% of the overall population. However, only 1 week would be required to vaccinate individuals working in critical infrastructure sectors (Tier 1 and 2), three weeks for Tier 3, two months for Tier 4, about 2 months for Tier 5, and 1 month for Tier 6 (Fig. 4, and Additional file 1: Fig. S1). With an expected 0.61 billion doses produced this year [10], and given a fixed 70% uptake rate among tiers, the estimated vaccine supply could cover individuals in Tiers 1–3 and half of individuals in Tier 4 given a two-dose vaccination schedule.

Sensitivity analyses show it will take 8 months to vaccinate 90% of individuals given 10 million doses administered each day; 1.8 years to vaccinate 70% of individuals given 3 million doses administered each day; 2.3 years to vaccinate 90% of individuals given 3 million doses administered each day (Additional file 1: Figs. S3-S5). It will take around 4 months to vaccinate 90% and 70% of individuals respectively, if the capacity of COVID-19 vaccination delivery was scaled up to 20 million doses administered each day (Additional file 1: Figs. S6-S7).

## Discussion

In the absence of specific antiviral treatment for COVID-19, vaccination likely represents the most promising way to control the COVID-19 pandemic. However, even if a COVID-19 vaccine becomes available, initial supplies will inevitably be limited. Supply issues could persist in the long term, due to huge global demand and limited production capacity. Almost everyone can potentially benefit from vaccination because of residual high susceptibility to SARS-CoV-2 infection. Considering different goals of a future vaccination program, changes in vaccine supplies, various levels of responsibility of population groups to the COVID-19 pandemic responses and essential services, as well as the risk of severe outcome and illness, we recommend a phased universal COVID-19 vaccination program for mainland China. Workers in critical sectors, including healthcare workers, law enforcement and security personnel, personnel in nursing home, and social welfare institutes, as well as sectors of energy, water, food, and transportation, and overseas workers/students (49.7 million) are the main candidates to receive high priority for vaccination, in order to maintain essential societal functions. Subsequently, we propose to extend the vaccination program to older adults, pregnant women, and those with underlying medical conditions (563.6 million), in order to reduce severe outcomes of COVID-19. Finally, working-age adults, school-age children, and younger children (784.8 million) could be vaccinated in order to reduce symptomatic COVID-19 infections, and/or to stop SARS-CoV-2 transmission.

Target population groups are further grouped into vaccination tiers from 1 to 6, with Tier 1 having the highest priority. Even though individuals within a tier have equal priority for vaccination, it may be necessary to sub-prioritize vaccination of groups within a tier if initial vaccine supplies are severely limited. For instance, cold-chain workers who have been particularly affected by COVID-19 and are often linked to workplace transmission could thus be vaccinated before other personnel within Tier 2 [70]. Other examples are represented by individuals aged ≥ 80 years or older with underlying conditions, who may be vaccinated before other personnel within Tier 3 or by individuals of < 60 years of age with ≥ 2 underlying conditions who may represent a sub-prioritized category within their Tier [20]. Further studies are warranted to examine the sub-prioritization within each vaccination tier. Although other factors like smoking, being male, and being an ethnic minority were found to be risk factors of severe outcome and deaths from COVID-19 in previous studies [71–73], they were not accounted for when determining priority population here due to consideration of equity and feasibility of vaccination.

The Joint Committee on Vaccination and Immunisation (JCVI) in the UK largely prioritizes individuals for vaccination based on age, considering simple age-based programs to be easier to implement and thus have a higher chance of achieving a high vaccine uptake [74]. As of December 1, 2020, JCVI does not provide precise advice on the prioritization for frontline healthcare and social workers. On the other hand, the Framework for Equitable Allocation of COVID-19 Vaccine of the National Academy of Sciences, Engineering, and Medicine suggests that in the US priority should be given to frontline healthcare workers, and those having significant risk of severe illness or death from COVID-19 (as individuals with two or more underlying health conditions) [20]. Compared to the UK and the US, the epidemiological situation in China is quite different, with an almost entirely susceptible population to SARS-CoV-2 infection and very limited local transmission. In this context, the frontline workers and individuals studying/working abroad represent the categories at higher risk of infections in mainland China. Our advice on priority populations for a COVID-19 vaccination came under the umbrella of the WHO SAGE Values Framework for The Allocation and Prioritization of COVID-19 Vaccination [5], and took into consideration the local context and the possible goals of a COVID-19 vaccination program in China.

The majority of the current COVID-19 vaccine candidates are being trialed as two-dose schedules [9]. A total of 70 million, 789 million, and 1099 million doses are separately needed to cover 70% of individuals in critical infrastructure sectors, persons at high risk of severe outcomes of COVID-19, and persons at high risk of acquiring symptomatic illness/infections. Between 2007 and 2015, the volume of all vaccines supplied (*n* = 55) licensed in mainland China varied from 666 million doses to 1.19 billion doses per year [75]. Several manufacturers state that a total of 0.61 billion doses of COVID-19 vaccine could be produced this year and 2.1 billion doses in 2021 [10]. Even if these candidate vaccines could be licensed and manufactured smoothly, it will take 7 months to vaccinate 70% of the general population. This is assuming an optimistic vaccine delivery rate that is over twofold higher than the maximum rate at which H1N1pdm vaccines were delivered in 2009 (3 million doses administered each day). Such a large-scale vaccination program like COVID-19 could also represent a major challenge for current the National Immunization Program in China, which is currently focused on childhood vaccination rather than on adult vaccination. The limited production capacity will likely further delay COVID-19 vaccination programs. This dilemma is likely not unique to China, and other countries across the world, particularly in low- and middle-income regions, will face a similar challenge.

Although according to survey results [13–15], 72.5–91.3% of the Chinese population aged 18 years or above appear to be willing to accept a COVID-19 vaccine, specific groups like pregnant women may be less willing to get COVID-19 vaccine due to safety concerns. These factors may delay or reduce the effective vaccine coverage. The acceptance of COVID-19 vaccination in specific segments of the population merit further studies.

Identifying individuals with underlying conditions is critical for a risk-based vaccination campaign. In China, the National Basic Public Health Service Program provides all residents with electronic health records, which have information on underlying conditions and can be queried by community healthcare centers [76]. Electronic health records as well as other medical records may be used to identify high-risk individuals with underlying conditions.

Our study has a number of limitations. First, we have qualitatively discussed the segments of the population to be prioritized in a COVID-19 vaccination program as well as the rationale behind prioritization choices. However, we could not quantitatively examine whether prioritizing older adults to reduce severe outcomes is a better choice than prioritizing working-age adults or school-age children to reduce illness/transmission. Mathematical modeling is urgently needed to assess both the health and economic impacts of potential vaccination strategies, and the potential to reduce for herd immunity benefits. Second, we did not consider eligibility for vaccination due to lack of efficacy and/or safety concerns that may affect specific groups such as older adults, people with pre-existing medical conditions, pregnant women, and very young children, since no vaccine has been licensed yet. Third, we did not consider real-time reactive outbreak immunization strategies because it is impossible to estimate the corresponding target population size. However, we strongly recommend use of COVID-19 vaccination during local outbreaks coupled with other non-pharmaceutical interventions in order to prevent subsequent waves of disease. Moreover, we did not discuss prioritization based on geography; the risk of COVID-19 exposure may be low in regions that have seen widespread COVID-19 activity by the time the vaccine is available and have a high level of population immunity. This may not be particularly relevant for China where the epidemic has been well controlled, but it may affect vaccine prioritization in other regions.

Because of the high burden and limited capacity for vaccine production, we have highlighted that more attention should be paid to low- and middle-income countries. The WHO SAGE Values Framework for The Allocation and Prioritization of COVID-19 Vaccination offers guidance for allocating and targeting COVID-19 pandemic vaccines, by providing six core principles and twelve objectives that further specify the six principles [5]. We tailored it to China-specific contexts accounting for the risk of illness and transmission, lessons learned from the response to the COVID-19 outbreak in Wuhan, the objectives of COVID-19 pandemic responses, and experience gained from the 2009 H1N1 pandemic vaccination program in China, in addition to the risk of severe outcomes, symptomatic illness, and transmission. Our recommendations for mainland China could be used as a template for usage of such guidelines. When a vaccine becomes available, our recommendations need to be reassessed to consider the eligibility of population subgroups based on the licensure label. They also need to be further reassessed periodically to account for changes in vaccine supply, demand, and local epidemiology. Although we propose a general framework to define vaccination priorities, the proposed vaccination program needs to be tailored locally, accounting for country-specific contexts such the objectives of the pandemic responses, the local level of transmission, the make-up of first responders, and essential workers as well as the capacity of immunization services.

## Conclusions

Vaccine deployment is likely to become vitally important for the global response to the COVID-19 pandemic. Here we provide a general framework to define priority groups for a phased introduction of a universal COVID-19 vaccination program. We applied this framework to mainland China and further estimated the corresponding target population sizes. The proposed vaccination program could assist Chinese policy-makers in the roll-out of a large-scale immunization program and be used as a reference for other countries, especially in low- and middle-income regions.

## Supplementary Information

**Additional file 1: Table S1.** COVID-19 vaccine priority groups. **Figure S1.** Schematic diagram of vaccinating 70% of target populations by Tiers. **Table S2.** Characteristics of systematic reviews for persons at high risk of severe outcome of COVID-19. **Table S3.** Age distribution of COVID-19 cases. **Table S4.** Data used to estimate the COVID-19 incidence rate. **Figure S2**. Pooled incidence of COVID-19 cases, stratified by age. **Figure S3.** Sensitivity analyses on days needed to vaccinate 90% of the target population, under the assumption that 10 million doses are administered per day. **Figure S4.** Sensitivity analyses on days needed to vaccinate 70% of the target population, under the assumption that 3 million doses are administered per day. **Figure S5.** Sensitivity analyses on days needed to vaccinate 90% of the target population, under the assumption that 3 million doses are administered per day. **Figure S6.** Sensitivity analyses on days needed to vaccinate 70% of the target population, under the assumption that 20 million doses are administered per day. **Figure S7.** Sensitivity analyses on days needed to vaccinate 90% of the target population, under the assumption that 20 million doses are administered per day.

## Data Availability

All data and data source were provided in details on GitHub at https://github.com/wenzidebanxia/target_population*.*

## Abbreviations

COVID-19: Coronavirus disease 2019
WHO: World Health Organization
SAGE: Strategic Advisory Group of Experts on Immunization
SARS-CoV-2: Novel severe acute respiratory syndrome coronavirus 2
US: United States
UK: United Kingdom
GBD: Global Burden of Diseases, Risk Factors, and Injuries Study
UN: United Nations
VE: Vaccine efficacy
*R*_*0*_: Basic reproduction number
H1N1pdm: Pandemic influenza H1N1
JCVI: The Joint Committee on Vaccination and Immunisation
