# Supplementary material for: Who should be prioritized for COVID-19 vaccination in China? A descriptive study
Source: BMC Med. 2021 Feb 10;19:45. doi: 10.1186/s12916-021-01923-8 (PMC7872877; doi:10.1186/s12916-021-01923-8)
Supplement: Supplementary file 1 — Additional file 1: Table S1. COVID-19 vaccine priority groups. Figure S1. Schematic diagram of vaccinating 70% of target populations by Tiers. Table S2. Characteristics of systematic reviews for persons at high risk of severe outcome of COVID-19. Table S3. Age distribution of COVID-19 cases. Table S4. Data used to estimate the COVID-19 incidence rate. Figure S2. Pooled incidence of COVID-19 cases, stratified by age. Figure S3. Sensitivity analyses on days needed to vaccinate 90% of the target population, under the assumption that 10 million doses are administered per day. Figure S4. Sensitivity analyses on days needed to vaccinate 70% of the target population, under the assumption that 3 million doses are administered per day. Figure S5. Sensitivity analyses on days needed to vaccinate 90% of the target population, under the assumption that 3 million doses are administered per day. Figure S6. Sensitivity analyses on days needed to vaccinate 70% of the target population, under the assumption that 20 million doses are administered per day. Figure S7. Sensitivity analyses on days needed to vaccinate 90% of the target population, under the assumption that 20 million doses are administered per day. [file 12916_2021_1923_MOESM1_ESM.docx]

**Supplementary information**

**Who should be prioritized for COVID-19 vaccination in China? A descriptive study**

Juan Yang, PhD ^1^, Wen Zheng, BSc ^1^, Huilin Shi, BSc ^1^, Xuemei Yan, BSc ^1^, Kaige Dong, BSc ^1^, Qian You, BSc ^1^, Guangjie Zhong, BSc ^1^, Hui Gong, BSc ^1^, Zhiyuan Chen, BSc ^1^, Mark Jit, PhD ^2,3,4^, Cecile Viboud, PhD^5^, Marco Ajelli, PhD^6,7^, Prof Hongjie Yu, PhD^1,8,9*^

*Correspondence: yhj@fudan.edu.cn

School of Public Health, Fudan University, Key Laboratory of Public Health Safety, Ministry of Education, Shanghai, China

**Affiliations:**

1. School of Public Health, Fudan University, Key Laboratory of Public Health Safety, Ministry of Education, Shanghai, China

2. Centre for Mathematical Modelling of Infectious Diseases, London School of Hygiene and Tropical Medicine, London, United Kingdom

3. Department of Infectious Disease Epidemiology, London School of Hygiene & Tropical Medicine, London, United Kingdom

4. WHO Collaborating Centre for Infectious Disease Epidemiology and Control, School of Public Health, Li Ka Shing Faculty of Medicine, The University of Hong Kong, Hong Kong Special Administrative Region, China

5. Division of International Epidemiology and Population Studies, Fogarty International Center, National Institutes of Health, Bethesda, MD, USA

6. Department of Epidemiology and Biostatistics, Indiana University School of Public Health, Bloomington, IN, USA

7. Laboratory for the Modeling of Biological and Socio-technical Systems, Northeastern University, Boston, MA, USA

8. Shanghai Institute of Infectious Disease and Biosecurity, Fudan University

9. Department of infectious diseases, Huashan Hospital, Fudan University

[Additional file 1 4](#_Toc61357724)

[Table S1. COVID-19 vaccine priority groups 4](#_Toc61357725)

[Figure S1. Schematic diagram of vaccinating 70% of target populations by Tiers, given 10 million doses administered per day and a two-dose vaccination schedule with a three-week interval 8](#_Toc61357726)

[Table S2. Characteristics of systematic reviews for persons at high risk of severe outcome of COVID-19 9](#_Toc61357727)

[Table S3. Age distribution of COVID-19 cases 36](#_Toc61357728)

[Table S4. Data used to estimate the COVID-19 incidence rate 39](#_Toc61357729)

[Figure S2. Pooled incidence of COVID-19 cases, stratified by age. 40](#_Toc61357730)

[Figure S3. Sensitivity analyses on days needed to vaccinate 90% of the target population, stratified by vaccination tier, under the assumption that 10 million doses are administered per day. Note that values reported within the square denote 90% of the population size in each tier; m denotes million. 41](#_Toc61357731)

[Figure S4. Sensitivity analyses on days needed to vaccinate 70% of the target population, stratified by vaccination tier, under the assumption that 3 million doses are administered per day. Note that values reported within the square denote 70% of the population size in each tier; m denotes million. 42](#_Toc61357732)

[Figure S5. Sensitivity analyses on days needed to vaccinate 90% of the target population, stratified by vaccination tier, under the assumption that 3 million doses are administered per day. Note that values reported within the square denote 90% of the population size in each tier; m denotes million. 43](#_Toc61357733)

[Figure S6. Sensitivity analyses on days needed to vaccinate 70% of the target population, stratified by vaccination tier, under the assumption that 20 million doses are administered per day. Note that values reported within the square denote 70% of the population size in each tier; m denotes million. 44](#_Toc61357734)

[Figure S7. Sensitivity analyses on days needed to vaccinate 90% of the target population, stratified by vaccination tier, under the assumption that 20 million doses are administered per day. Note that values reported within the square denote 90% of the population size in each tier; m denotes million. 45](#_Toc61357735)

[References 46](#_Toc61357736)

# Additional file 1

# Table S1. COVID-19 vaccine priority groups

|  | WHO[1] | US[2] | UK[3] |
| --- | --- | --- | --- |
| Developed by | Strategic Advisory Group of Experts on Immunization | Committee on Equitable Allocation of Vaccine for the Novel Coronavirus | Joint Committee on Vaccination and Immunisation |
| Published date | 14 September 2020 | 2 October 2020 | 25 September 2020 |
| Underlying principles of the advice on prioritization of COVID-19 vaccination | Human well-being, equal respect, global equity, national equity, reciprocity and Legitimacy | 1. Ethical Principles: Maximum Benefit, Equal Concern, and Mitigation of Health Inequities  2. Procedural Principles: Fairness, Transparency, and Evidence-Based | To reduce mortality, improve population health by reducing serious disease, and to protect the NHS and social care system |
| Approaches to set general priorities | Considering both specific value principles and objectives, including but not limited to reducing deaths and disease burden from the COVID-19 pandemic, and reducing societal and economic disruption (other than through reducing deaths and disease burden) | Using four risk-based criteria to set general priorities: (1) risk of acquiring infection, (2) risk of severe morbidity and mortality, (3) risk of negative societal impact, and (4) risk of transmitting infection to others | Considering both clinical risk stratification and an age-based approach, but largely prioritizes based on age |
| Ranking of prioritization | The document explicitly connects priority groups with specific value principles and objectives (examples shown below). Given country-specific nuances in epidemiology, demographics, and vaccine delivery systems, these priority groups will need to be further interpreted at a national level. Country-level decision making will require data collected, or at least collated, at the country-level.  ***1. To reduce deaths and disease burden***  (1) Populations with significantly elevated risk of severe disease or death  • Older adults defined by age-based risk  • Older adults in high risk living situations  • Groups with comorbidities or health states (e.g. pregnancy/lactation) determined to be at significantly higher risk of severe disease or death  • Sociodemographic groups at disproportionately higher risk of severe disease or death  (2) Populations with significantly elevated risk of being infected  • Health workers at high or very high risk  • Employment categories unable to physically distance  • Social groups unable to physically distance  • Groups living in dense urban neighborhoods  • Groups living in multigenerational households  ***2. To reduce societal and economic disruption***  • Age groups at high risk of transmitting SARS-CoV-2  • Non age-based population groups with significantly elevated risk of infection and transmission  • School-aged children to minimize disruption of education and socioemotional development  • Groups targeted as part of an emergency outbreak response using emergency vaccine reserves  • Workers in non-essential but economically critical sectors, particularly in occupations that do not permit remote work or physical distancing while working | A four-phased approach:  ***1. Phase 1***   - 1a: “frontline” health workers; first responders - 1b: People of all ages with two or more comorbid and underlying conditions; older adults living in congregate or overcrowded settings   ***2. Phase 2***  • K–12 teachers and school staff and child care workers  • Critical workers who are in industries essential to the functioning of society and at substantially higher risk of exposure  • People of all ages with one comorbid and underlying condition  • People in homeless shelters or group homes for individuals with disabilities, including serious mental illness, developmental and intellectual disabilities, and physical disabilities or in recovery, and staff who work in such settings  • People in prisons, jails, detention centers, and similar facilities, and staff who work in such settings  • All older adults not included in Phase 1  ***3. Phase 3***  • Young adults  • Children  • Workers in industries and occupations important to the functioning of society and at increased risk of exposure not included in Phase 1 or 2  ***4. Phase 4***  • Everyone residing in the United States who did not have access to the vaccine in previous phases | 1. Older adults’ resident in a care home and care home workers  2. All those 80 years of age and over and health and social care workers  3. All those 75 years of age and over  4. All those 70 years of age and over  5. All those 65 years of age and over  6. High-risk adults under 65 years of age  7. Moderate-risk adults under 65 years of age  8. All those 60 years of age and over  9. All those 55 years of age and over  10. All those 50 years of age and over  11. Rest of the population (priority to be determined) |

#

# Figure S1. Schematic diagram of vaccinating 70% of target populations by Tiers, given 10 million doses administered per day and a two-dose vaccination schedule with a three-week interval

Numbers in the colored boxes denote the accumulative days to vaccinate the target population in a separate Tier. Note: overlaps of vaccination exist between adjacent tiers. For instance, there will be 20 “blank days” to vaccinate Tier 1 population due to a three-week interval between two doses administered. To avoid wastage, Tier 2 and 3 could be vaccinated in these “blank days”.

# Table S2. Characteristics of systematic reviews for persons at high risk of severe outcome of COVID-19

| **No.** | **Author** | **Title** | **Journal** | **Pulished date** | **Database** | **End date of included articles** | **Papers included in meta-analysis** | **Sample size included in meta-analysis** | **Rating scale** | **Quality of included articles** |
| --- | --- | --- | --- | --- | --- | --- | --- | --- | --- | --- |
| 1 | Zheng Z, Peng F, Xu B[4] | Risk factors of critical & mortal COVID-19 cases: A systematic literature review and meta-analysis | J Infect | 2020/4/23 | Pubmed, Embase, Web of Science, and CNKI | 2020/3/20 | 13 | 3027 | MINORS | The global ideal score being 24 for comparative studies. None of the studies was considered to be seriously flawed according to the MINORS assessment. The 13 included studies scored between 18 and 21. All studies were considered to have a low risk of bias for selection. |
| 2 | Singh AK, Gillies CL, Singh R[5] | Prevalence of comorbidities and their association with mortality in patients with COVID-19: A Systematic Review and Meta-analysis | Diabetes Obes Metab | 2020/6/23 | Medline, Scopus and the World Health Organisation (WHO) website | 2020/4/23 | 18 | 14,558 | NOS | All studies scored at least seven, with the majority scoring eight |
| 3 | Wang X, Fang X, Cai Z[6] | Comorbid Chronic Diseases and Acute Organ Injuries Are Strongly Correlated with Disease Severity and Mortality among COVID-19 Patients: A Systemic Review and Meta-Analysis | Research | 2020/4/19 | PubMed, Embase, Web of Science, medRxiv, and bioRxiv | 2020/4/6 | 34 | 6,263 | No |  |
| 4 | Taylor E, Hofmeyr R, Torborg A[7] | Risk factors and interventions associated with mortality or survival in adult covid-19 patients admitted to critical care: A systematic review and meta-analysis | South Afr J Anaesth Analg |  | MEDLINE (PubMed), CINAHL (via EBSCOHOST) Scopus (including Embase), Web of Science (all databases), Cochrane Central Register of Controlled Trials, and Proquest | 2020/4/7 | 9 | 1853 | mNOS | Overall, one study was of a high methodological quality with a score of 7 stars. The remaining eight included studies were considered to be of low methodological quality. |
| 5 | Wang B, Li R, Lu Z[8] | Does comorbidity increase the risk of patients with COVID-19: evidence from meta-analysis | Aging-Us | 2020/4/8 | PubMed, Cochrane Library, Embase, Springer, Web of Science | 2020/3/1 | 6 | 1558 | NOS | All articles are of high quality because of NOS score no less than 6 |
| 6 | Jain V, Yuan JM[9] | Predictive symptoms and comorbidities for severe COVID-19 and intensive care unit admission: a systematic review and meta-analysis | Int J Public Health | 2020/5/25 | MEDLINE, EMBASE and Global Health | 2020/3/5 | 7 | 1813 | STROBE | Each paper was assigned an overall quality score based on the percentage of STROBE checklist criteria met (< 55% = −, 55–65% = +, > 65% = ++). Four articles are +, one is -, and two are ++. |
| 7 | Giannakoulis VG, Papoutsi E, Siempos I[10] | Effect of cancer on clinical outcomes of patients with COVID-19: A meta-analysis of patient data | JCO Glob Oncol | 2020/6/8 | PubMed, medRxiv, COVID-19 Open Research Dataset (CORD-19) | 2020/4/27 | 32 | 46499 | Tool to Assess Risk of Bias in Cohort Studies | Five of the articles are low risk of bias |
| 8 | Tian, W, Jiang, W, Yao J[11] | Predictors of mortality in hospitalized COVID-19 patients: A systematic review and meta-analysis | J Med Virol | 2020/5/22 | PubMed, Google scholar, Web of Science, CNKI | 2020/4/24 | 14 | 4659 | AHRQ | 8 is high, 5 is moderate, 1 is low |
| 9 | Li J, He X, Yuan Y[12] | Meta-analysis investigating the relationship between clinical features, outcomes, and severity of severe acute respiratory syndrome coronavirus 2 (SARS-CoV-2) pneumonia | Am J Infect Control | 2020/6/12 | PubMed, Embase, Cochrane Library, medRxiv | 2020/4/14 | 12 | 2445 | NOS | All articles are no less than 6 |
| 10 | Wu Z, Tang Y , Cheng Q[13] | Diabetes increases the mortality of patients with COVID-19: a meta-analysis | Acta Diabetol | 2020/6/24 | Medline via PubMed, EMBASE, Web of Science | 2020/4/14 | 9 | 1471 | NOS | All articles are of high quality because NOS score was no less than 6. |
| 11 | Xu L, Mao Y, Chen G[14] | Risk factors for 2019 novel coronavirus disease (COVID-19) patients progressing to critical illness: a systematic review and meta-analysis | Aging-Us | 2020/6/23 | CNKI, Wanfang Database, Weipu Database, Chinese Biomedicine Literature Database (CBM-SinoMed), PubMed, Embase, Cochrane Central Register and Web of Science | 2020/3/8 | 20 | 4062 | AHRQ | All studies contained complete data sources, inclusion and exclusion criteria, and reasonable control of confounding factors. Nonetheless, only a few studies reported their quality control and management of missing data |
| 12 | Ssentongo P, Ssentongo AE, Heilbrunn ES[15] | Association of cardiovascular disease and 10 other pre-existing comorbidities with COVID-19 mortality: A systematic review and meta-analysis | PLoS One | 2020/8/26 | MEDLINE, OVID, SCOPUS, Joana Briggs International EBP, Cochrane Library databases, Google Scholar, Medrxiv | 2020/7/17 | 25 | 65484 | NOS | Median quality score was 7 (range = 5–9) |
| 13 | Bellou V, Tzoulaki I, Evangelou E[16] | Risk factors for adverse clinical outcomes in patients with COVID-19: A systematic review and meta-analysis | medRxiv | 2020/5/19 | PubMed | 2020/4/19 | 88 | more than 1000 | No |  |
| 14 | Rahman A, Sathi NJ[17] | Risk factors of the severity of COVID-19: A meta-analysis | medRxiv | 2020/5/10 | PubMed, Google Scholar, EMBASE, and Cochrane Library | 2020/4/18 | 10 | 2272 | No |  |
| 15 | Khan M, Khan MN, Mustagir MG[18] | Effects of underlying morbidities on the occurrence of deaths in COVID-19 patients: A systematic review and meta-analysis | J Glob Health | 2020/8/18 | Medline, Web of Science, Scopus, CINAHL; reference list of the selected studies, relevant journal websites, and renowned pre-print servers (medRxiv, bioRxiv, SSRN) | 2020/5/1 | 35 | 25031 | NOS | 1 study scored 9, 10 studies scored 8, 10 studies scored 7, 13 studies scored 6, 4 studies scored 5 |
| 16 | Soeroto AY, Soetedjo NN, Purwiga A[19] | Effect of increased BMI and obesity on the outcome of COVID-19 adult patients: A systematic review and meta-analysis | Diabetes Metab Syndr | 2020/9/28 | PubMed,Embase | 2020/7/28 | 16 | 6690 | No |  |
| 17 | Liu Y, Zhang Z, Pan X[20] | The chronic kidney disease and acute kidney injury involvement in COVID-19 pandemic: A systematic review and meta-analysis | medRxiv | 2020/5/2 | PubMed, medRxiv, CNKI, WanFang | 2020/4/13 | 36 | 6395 | NOS | The NOS scores≥6 were considered high quality studies. The majority of included studies were poor quality except four studies whose NOS scores are 6. The NOS scores of other 31 trials are 5, and only one trial’s NOS scores were 4. |
| 18 | Matsushita K, Ding N, Kou M[21] | The relationship of COVID-19 severity with cardiovascular disease and its traditional risk factors: A systematic review and meta-analysis | Glob Heart | 2020/9/22 | PubMed, Embase | 2020/4/3 | 25 | 76638 | NOS | The NOS score for cohorts studies ranges from 0 to 9; a score greater than 6 was considered high-quality. Most of these publications were considered high quality |
| 19 | Tabrizi R, Lankarani KB, Nowrouzi-sohrabi P[22] | The role of comorbidities and clinical predictors of severe disease in COVID-19: a systematic review and meta-analysis | medRxiv |  | PubMed, Scopus, EMBASE, Web of Science, Google Scholar | 2020/3/12 | 17 | 3921 | NOS | We considered the quality assessment threshold with a NOS scored ≥ 7 being defined as good quality. Most paper's NOS score is over 5, except one paper. |
| 20 | Dorjee K, Kim H[23] | Epidemiological risk factors associated with death and severe disease in patients suffering from COVID-19：A comprehensive systematic review and meta-analysis | medRxiv | 2020/6/20 | Medline, Embase,Web of Science and the WHO COVID-19 database | 2020/5/22 | 44 | 20594 | NOS |  |
| 21 | Hessami A, Shamshirian A, Heydari K[24] | Cardiovascular diseases and COVID-19 mortality and intensive care unit admission: A systematic review and meta-analysis | medRxiv | 2020/4/16 | PubMed, Embase, Cochrane Library, Scopus, Web of Science,medRxiv, Science Direct search engine, journal websites | 2020/3/31 | 16 | 3473 | NOS | According to NOS tool for quality assessment, 16 studies earned the minimum eligibility score and entered into the meta-analysis for primary outcomes |
| 22 | Chen Y, Gong X, Wang L[25] | Effects of hypertension, diabetes and coronary heart disease on COVID-19 diseases severity: a systematic review and meta-analysis | medRxiv | 2020/3/30 | PubMed, Web of Science, China National Knowledge Infrastructure, WanFang and VIP | 2020/3/6 | 9 | 1936 | NOS | Except for 1 scored 5 points, other studies scored 6 points and above. |
| 23 | Parohan M, Yaghoubi S, Seraji A[26] | Risk factors for mortality in patients with Coronavirus disease 2019 (COVID-19) infection: a systematic review and meta-analysis of observational studies | Aging Male | 2020/6/8 | Web of Science, PubMed, Scopus, Cochrane Library and Google scholar | 2020/5/1 | 14 | 31,354 | NOS | In the present study, articles with the NOS score of 5 were considered as high quality publications |
| 24 | Ma C, Gu J, Hou P[27] | Incidence, clinical characteristics and prognostic factor of patients with COVID-19 a systematic review and meta-analysis | medRxiv | 2020/3/20 | PubMed, the Web of Science Core Collection, Embase, Cochrane and MedRxiv | 2020/2/19 | 30 | 53000 | AHRQ | 6 studies scored 10, 12 study scored 9, 5 studies scored 8, 3 studies scored 7, 3 studies scored 6, 1 study scored 5 |
| 25 | Palaiodimos L, Chamorro-Pareja N, Karamanis D[28] | Diabetes is associated with increased risk for in-hospital mortality in patients with COVID-19: a systematic review and meta-analysis comprising 18,506 patients | Hormones (Athens) | 2020/10/29 | Medline, Embase, Google Scholar, and medRxiv | 2020/5/10 | 14 | 18,506 | QUIPS | All the studies were found to have a low risk of bias |
| 26 | Jutzeler CR, Bourguignon L, Weis CV[29] | Comorbidities, clinical signs and symptoms, laboratory findings, imaging features, treatment strategies, and outcomes in adult and pediatric patients with COVID-19 A systematic review and meta-analysis | Travel Med Infect Dis | 2020/8/4 | EMBASE, PubMed/Medline, Scopus, and Web of Science | 2020/3/28 | 148 | 12,149 | No |  |
| 27 | Islam MS, Barek MA, Aziz MA[30] | Association of age, sex, comorbidities, and clinical symptoms with the severity and mortality of COVID-19 cases a meta-analysis with 85 studies and 67299 cases | medRxiv | 2020/5/26 | PubMed, ScienceDirect, SAGE | 2020/5/17 | 85 | 67,299 | NOS | only two studies being of moderate quality (score 5), others were of high quality (score ranges between 6-8) |
| 28 | Zaki N, Mohamed EA, Ibrahim S[31] | The influence of comorbidity on the severity of COVID-19 disease systematic review and analysis | medRxiv | 2020/6/20 | COVID-19 Open Research Dataset (CORD-19),PubMed, Europe PMC, and Google Scholar | 2020/5/20 | 12 | 4101 | No |  |
| 29 | Cao G, Li P, Cheng Y[32] | A Risk Prediction Model for Evaluating the disease progression of COVID-19 pneumonia based on Meta-analysis and 214 Clinical Cases | SSRN |  | PubMed, Web of Science, and CNKI | 2020/3/18 | 32 | 6061 | NOS | no publication bias exists |
| 30 | Salunke AA, Nandy K, Pathak SK[33] | Impact of COVID -19 in cancer patients on severity of disease and fatal outcomes: A systematic review and meta-analysis | Diabetes Metab Syndr |  | PubMed, Cochrane Central Register of Clinical Trials | 2020/4/16 | 13 | 3775 | NOS |  |
| 31 | Sreenivasan J, Khan MS, Anker SD[34] | Cardiovascular Risk Factors and Complications in Patients Infected with COVID-19: A Systematic Review | SSRN |  | MEDLINE, SCOPUS and EMBASE | 2020/3/30 | 10 | 1,427 | New Castle Ottawa Scale | The majority of the studies had moderate-to-high risk of bias because of retrospective study design and lack of extended follow up |
| 32 | Biswas M, Rahaman S, Biswas TK[35] | Effects of Sex, Age and Comorbidities on the Risk of Infection and Death Associated with COVID-19: A Meta-Analysis of 47807 Confirmed Cases | SSRN |  | PubMed, Cochrane Library and Scinapse, important journal websites (New England Journal of Medicine; Journal of American Medical Association; Lancet; Nature; British Medical Journal | 2020/3/24 | 21 | 47,807 | NOS | The quality of the majority of included studies as assessed by the Newcastle Otawa scale was of high quality (score ranges etween 6-8) with only one study was of moderate quality (score 5) |
| 33 | Kumar A, Arora A, Sharma P[36] | Clinical Features of COVID-19 and Factors Associated with Severe Clinical Course: A Systematic Review and Meta-analysis | SSRN | 2020/4/21 | PubMed | 2020/3/17 | 21 | 3496 | the National Institute of Health (NIH) tools | all the included studies were of good quality. |
| 34 | Aggarwal G, Cheruiyot I, Aggarwal S[37] | Association of Cardiovascular Disease With Coronavirus Disease 2019 (COVID-19) Severity: A Meta-Analysis | Curr Probl Cardiol | 2020/5/14 | Pubmed, Embase, and Cochrane Central Register of Controlled Trials (CENTRAL) | 2020/4/20 | 18 | 4858 | NOS |  |
| 35 | Tian Y, Qiu X, Wang C[38] | Cancer associates with risk and severe events of COVID-19: A systematic review and meta-analysis | International journal of cancer | 2020/7/20 | PubMed, Elsevier, Web of Science,CNKI, WanFang, VIP | 2020/4/23 | 38 | 7,094 | No |  |
| 36 | Guo L, Shi Z, Zhang Y[39] | Comorbid diabetes and the risk of disease severity or death among 8807 COVID-19 patients in China: A meta-analysis | Diabetes Res Clin Pract | 2020/7/28 | PubMed, Web of Knowledge, medRxiv, bioRxiv, CNKI, Wanfang | 2020/5/30 | 9 | 8807 | No |  |
| 37 | Zhou Y, Yang Q, Chi J[40] | Comorbidities and the risk of severe or fatal outcomes associated with coronavirus disease 2019: A systematic review and meta-analysis | nt J Infect Dis | 2020/7/30 | PubMed, EMBASE, and the Cochrane Library | 2020/4/25 | 34 | 16110 | No |  |
| 38 | Mantovani A, Byrne CD, Zheng MH, Targher G[41] | Diabetes as a risk factor for greater COVID-19 severity and in-hospital death: A meta-analysis of observational studies | Nutr Metab Cardiovasc Dis | 2020/6/24 | PubMed, Scopus and Web of Science | 2020/5/15 | 83 | 78,874 | NOS | 11 studies scored 6, others scored 5 |
| 39 | Fang X, Li S, Yu H[42] | Epidemiological, comorbidity factors with severity and prognosis of COVID-19 a systematic review and meta-analysis | Aging (Albany NY) | 2020/7/14 | Pubmed, medRxiv, bioRxiv | 2020/4/5 | 61 | 15071 | NOS | The NOS score ranged from 5 to 7, which   means a moderate methodological quality |
| 40 | Li X, Guan B, Su T[43] | Impact of cardiovascular disease and cardiac injury on in-hospital mortality in patients with COVID-19: a systematic review and meta-analysis | Heart | 2020/5/29 | PubMed, Embase and Web of Science | 2020/4/14 | 10 | 3118 | NOS | The studies with 7 points or more were considered of high quality. 8 studies scored 7 or more, 2 studies scored 6. |
| 41 | Pranata R, Huang I, Lim MA[44] | Impact of cerebrovascular and cardiovascular diseases on mortality and severity of COVID-19-systematic review, meta-analysis, and meta-regression | Journal of stroke and cerebrovascular diseases : the official journal of National Stroke Association | 2020/5/16 | PubMed, SCOPUS, EuropePMC, and Cochrane Central Database | 2020/4/10 | 16 | 4448 | No |  |
| 42 | Hussain A, Mahawar K, Xia Z, Yang W, El-Hasani S[45] | Obesity and mortality of COVID-19. Meta-analysis | Obes Res Clin Pract | 2020/7/15 | PubMed, Embase, Google, Google Scholar, and Springer, Elsevier, the Lancet, AMJ, BMJ, and Oxford journals | 2020/5/1 | 14 | 403535 | NOS | 4 studies scored 6 stars, 2 study scored 5 stars, 6 studies scored 4 stars, 2 studies scored 3 stars |
| 43 | Földi M, Farkas N, Kiss S[46] | Obesity is a risk factor for developing critical condition in COVID-19 patients: A systematic review and meta-analysis | Obesity reviews : an official journal of the International Association for the Study of Obesity | 2020/7/21 | Medline (via PubMed), Embase, Cochrane Central Register of Controlled Trials (CENTRAL), Scopus and Web of Science | 2020/5/11 | 10 | 3279 | QUIPS |  |
| 44 | Figliozzi S, Masci PG, Ahmadi N[47] | Predictors of Adverse Prognosis in Covid-19: A Systematic Review and Meta-analysis | Eur J Clin Invest | 2020/7/30 | PubMed/Medline and Scopus | 2020/4/24 | 51 | 29,653 |  | Six studies were adjudicated of fair quality while the remaining ones were considered of good quality |
| 45 | Kovalic AJ, Satapathy SK, Thuluvath PJ[48] | Prevalence of chronic liver disease in patients with COVID-19 and their clinical outcomes: a systematic review and meta-analysis | Hepatol Int | 2020/7/30 | MEDLINE/PubMed, EMBASE, and medRxiv | 2020/5/16 | 74 | 23,424 | No |  |
| 46 | Nandy K, Salunke A, Pathak SK[49] | Coronavirus disease (COVID-19): A systematic review and meta-analysis to evaluate the impact of various comorbidities on serious events | Diabetes Metab Syndr | 2020/7/8 | PubMed, Cochrane Central Register of Clinical Trials | 2020/4/28 | 16 | 3994 | NOS |  |
| 47 | Zhang J, Wu J, Sun X[50] | Association of hypertension with the severity and fatality of SARS-CoV-2 infection: A meta-analysis | Epidemiol Infect | 2020/5/29 | PubMed, Elsevier Science Direct, Web of science, Wiley Online Library and CNKI | 2020/3/20 | 18 | 4505 | NOS and STROBE | The methodological qualities of the included studies were similar. Nine studies had a moderate risk of participant comparability and confounding variables. Six, seven, and five studies had 6, 7, and 8 points of assessment score in the present analysis, respectively. In addition, no significant difference was observed between the results obtained from NOS and STROBE analysis |
| 48 | Huang I, Lim MA, Pranata R[51] | Diabetes mellitus is associated with increased mortality and severity of disease in COVID-19 pneumonia - A systematic review, meta-analysis, and meta-regression | Diabetes Metab Syndr | 2020/4/26 | PubMed and EuropePMC | 2020/4/8 | 30 | 6452 | No |  |
| 49 | Parveen R, Sehar N, Bajpai R[52] | Association of diabetes and hypertension with disease severity in covid-19 patients: A systematic literature review and exploratory meta-analysis | Diabetes Res Clin Pract | 2020/7/3 | PubMed, Medline, Cochrane and Google Scholar | 2020/3/31 | 7 | 2018 | NIH | The quality assessment indicated that most included studies were of acceptable quality.7篇中国 |
| 50 | Ofori-Asenso R, Ogundipe O, Agyeman AA[53] | Cancer is associated with severe disease in COVID-19 patients: a systematic review and meta-analysis | Ecancermedicalscience | 2020/6/23 | MEDLINE, EMBASE and ScienceDirect as well as websites of WHO and key public health institutions | 2020/4/28 | 20 | 32404 | NOS |  |
| 51 | Lu L, Zhong W, Bian Z[54] | A comparison of mortality-related risk factors of COVID-19, SARS, and MERS: A systematic review and meta-analysis | J Infect | 2020/7/4 | MEDLINE, EPISTEMONIKOS, COCHRANE, China National Knowledge Infrastructure and WANFANG STATA | 2020/4/11 | 10 | 11,818 | NOS | The included studies scored between 5 and 9. None of the studies was considered to be poor quality. |
| 52 | Liu M, Gao Y, Zhang Y[55] | The association between severe or dead COVID-19 and autoimmune diseases: A systematic review and meta-analysis | J Infect | 2020/6/6 | PubMed, Embase, Web of Science and Cochrane Library | 2020/5/8 | 6 | 2091 |  | The range of quality scores was 5 to 8, with a median of 7 (7.17 ±1.17) . |
| 53 | Liu H, Chen S, Liu M[56] | Comorbid Chronic Diseases are Strongly Correlated with Disease Severity among COVID-19 Patients: A Systematic Review and Meta-Analysis | Aging Dis | 2020/6/4 | PubMed, Ovid MEDLINE, EMBASE, CDC, NIH database and other sources such as Google, Google Scholar, and the AMED (Allied and Complementary Medicine) search engine | 2020/4/25 | 24 | 10948 | NOS | NOS scores of at lease six were considered high-quality literature. All scored 6 or more. |
| 54 | Lippi G, Wong J, Henry BM.[57] | Hypertension in patients with coronavirus disease 2019 (COVID-19): a pooled analysis | Pol Arch Intern Med | 2020/4/2 | Scopus, Medline, and Web of Science d | 2020/3/26 | 13 | 2893 | No |  |
| 55 | Aggarwal G, Lippi G, Lavie CJ[58] | Diabetes Mellitus Association with Coronavirus Disease 2019 (COVID-19) Severity and Mortality: A Pooled Analysis | Journal of diabetes | 2020/7/18 | PUBMED, EMBASE and Cochrane Central Register of Controlled Trials (CENTRAL) | 2020/3/31 | 16 | 3182 | No |  |
| 56 | Zhao Q, Meng M, Kumar R[59] | The impact of COPD and smoking history on the severity of COVID-19: A systemic review and meta-analysis | J Med Virol | 2020/4/16 | PubMed, Web of Science, Cochrane, WanFang Database, and CNKI | 2020/3/22 | 11 | 2002 | MINORS | The overall quality of available literature was moderate with MINORS scores ranging from 10 to 13 |
| 57 | Zhao J, Li X, Gao Y, Huang W.[60] | Risk factors for the exacerbation of patients with 2019 Novel Coronavirus: A meta-analysis | Int J Med Sci | 2020/7/6 | PubMed, Embase and Google scholar | 2020/2/8 | 2 | 179 | No |  |
| 58 | Yang J, Zheng Y, Gou X[61] | Prevalence of comorbidities and its effects in patients infected with SARS-CoV-2: a systematic review and meta-analysis | Int J Infect Dis | 2020/3/12 | PubMed, EMBASE, and Web of Science | 2020/2/25 | 7 | 1576 | No |  |
| 59 | Yang J, Hu J, Zhu C.[62] | Obesity aggravates COVID-19: a systematic review and meta-analysis | J Med Virol 2020 | 2020/7/1 | Pubmed, Embase, WOS, Cochrane, CNKI, Wanfang, and Sinomed | 2020/4/22 | 9 | 4444 | NOS | The quality of study design among the selected studies was median, with a median quality score of 6. |
| 60 | Wu J, Zhang J, Sun X[63] | Influence of diabetes mellitus on the severity and fatality of SARS-CoV-2 (COVID-19) infection | Diabetes Obes Metab | 2020/6/5 | PubMed, Embase, medRxiv, Cochrane library, and CNKI | 03/20/2020 | 12 | 2455 | NOS | All scored 6 or more. |
| 61 | Pranata R, Lim MA, Yonas E[64] | Body Mass Index and Outcome in Patients with COVID-19: A Dose-Response Meta-Analysis | Diabetes Metab | 2020/8/2 | PubMed, Europe PMC, ProQuest, and the Cochrane Central Database | 2020/5/28 | 12 | 34,390 | NOS | The mean NOS was 8.3 ± 1.1, indicating a low risk of bias. |
| 62 | Kumar A, Arora A, Sharma P[65] | Is diabetes mellitus associated with mortality and severity of COVID-19? A meta-analysis | Diabetes Metab Syndr | 2020/5/15 | PubMed | 2020/4/22 | 33 | 16003 | NIH | Thus 32 studies (97%) were judged as good quality and remaining 1 study (3%) was judged as fair quality (scores 6-7). None of the included study was judged poor. The single study with fair quality was the paper published by the CDC, USA on the COVID-19 cases reported to it from all over the US. Thus, it was a registry data, rather than a hospital-based study. |
| 63 | Alqahtani JS, Oyelade T, Aldhahir AM[66] | Prevalence, Severity and Mortality associated with COPD and Smoking in patients with COVID-19: A Rapid Systematic Review and Meta-Analysis | PloS one | 2020/5/12 | MEDLINE and Google scholar | 2020/3/24 | 15 | 2473 | NOS | The risk of bias ranged from 0.4 to 2.7; nine studies scored ≥ 2, which indicates low risk of bias |
| 64 | Liu M, Gao Y, Shi S, Chen Y, Yang K, Tian J[67] | Drinking no-links to the severity of COVID-19 a systematic review and meta-analysis | J Infect | 2020/5/28 | PubMed, EMBASE, Web of Science,CENTRAL, CNKI, CBM, Wanfang | 2020/5/8 | 6 | 1998 | No |  |
| 65 | Gao Y, Chen Y, Liu M, Shi S, Tian J[68] | Impacts of immunosuppression and immunodeficiency on COVID-19 A systematic review and meta-analysis | J Infect | 2020/5/15 | PubMed, EMBASE, Web of Science, CENTRAL, CNKI, CBM, Wanfang Database | 2020/4/25 | 8 | 4007 | No |  |
| 66 | Gao Y, Liu M, Chen Y, Shi S, Geng J, Tian J[69] | Association between tuberculosis and COVID‐19 severity and mortality A rapid systematic review and meta‐analysis | J Med Virol | 2020/7/21 | EMBASE, PubMed, Web of Science, the Cochrane Central Register of Controlled Trials (CENTRAL), Chinese Biomedical Literature Database (CBM),China National Knowledge Infrastructure (CNKI), and Wanfang | 2020/5/12 | 6 | 2765 | NOS | The included studies were rated six to eight stars according to the NOS scale |
| 67 | ElGohary GM, Hashmi S, Styczynski J[70] | The risk and prognosis of COVID-19 infection in cancer patients A systematic review and meta-analysis | Hematology/Oncology and Stem Cell Therapy | 2020/7/30 | Medline (via PubMed), Scopus, and Web of Science | 2020/5/27 | 22 | 11243 | No |  |
| 68 | Wu X, Liu L, Jiao J, Yang L, Zhu B, Li X[71] | Characterisation of clinical, laboratory and imaging factors related to mild vs. severe covid-19 infection a systematic review and meta-analysis | Annals of medicine | 2020/8/11 | Pubmed,Other databases | 2020/4/1 | 41 | 5064 | NOS | Quality assessment by the NOS indicates high-quality data for all including studies |
| 69 | Salunke AA, Nandy K, Pathak SK[33] | Impact of COVID -19 in cancer patients on severity of disease and fatal outcomes: A systematic review and meta-analysis | Diabetes Metab Syndr | 2020/7/28 | PubMed, Cochrane Central Register of Clinical Trials | 2020/4/16 | 13 | 3775 | NOS | our assessment of the studies included in the analysis shows that the quality of the evidence has been reasonably good. |
| 70 | Siepmann T, Sedghi A, Barlinn J[72] | Association of history of cerebrovascular disease with severity of COVID-19 | Journal of Neurology | 2020/8/6 | PubMed, EMBASE, and Cochrane Library | 2020/4/11 | 11 | 1805 | Oxford Centre for Evidence-based Medicine Rating Scale | All included studies from published literature were consistently graded as level of evidence 4 |
| 71 | Sanchez-Ramirez DC, Mackey D[73] | Underlying respiratory diseases, specifically COPD, and smoking are associated with severe COVID-19 outcomes A systematic review and meta-analysis | Respiratory Medicine | 2020/7/30 | PubMed, Web of Sciences, and Ovid MEDLINE | 2020/4/15 | 22 | 13184 | No |  |
| 72 | Wu T, Zuo Z, Kang S[74] | Multi-organ Dysfunction in Patients with COVID-19 A Systematic Review and Meta-analysis | Aging and disease |  | EMBASE, PubMed, Web of Science, MedRxiv, and Biorxiv | 2020/5/13 | 73 | 171108 | Agency for Healthcare Research and Quality | the quality of the majority of studies included in the meta-analysis was moderate or high |
| 73 | Pate U, Malik P, Shah D[75] | Pre-existing cerebrovascular disease and poor outcomes of COVID-19 hospitalized patients a meta-analysis | Journal of Neurology | 2020/8/8 | PubMed, Web of Science, and Scopus | 2020/4/30 | 11 | 4987 | Newcastle–Ottawa Quality Assessment Scale and Cochrane Collaboration’s tool | overall studies had moderate risk of bias |
| 74 | Sales-Peres SHdC, Azevedo-Silva LJd , Bonatoa RCS[76] | Coronavirus (SARS-CoV-2) and the risk of obesity for critically illness and ICU admitted Meta-analysis of the epidemiological evidence | Obesity Research & Clinical Practice | 2020/8/3 | PubMed, SCOPUS, Embase, BVS/LILACS, Web of Science, SCIELO, and Google Scholar | 2020/5/3 | 9 | 6577 | NOS | The quality assessment of studies using NOS indicated moderate quality, with scores ranging from 6 to 8. |
| 75 | Hariyanto TI, Kurniawan A[77] | Dyslipidemia is associated with severe coronavirus disease 2019 (COVID-19) infection | Diabetes & Metabolic Syndrome: Clinical Research & Reviews | 2020/8/1 | PubMed | 2020/7/9 | 7 | 6922 | No |  |
| 76 | Yin T, Li Y, Ying Y, Luo Z[78] | Association of Comorbidity with COVID-19 in Chinese Population: Analysis of Risk Factors of the COVID-19 Severity | SSRN |  | PubMed, Web of Science, and CNKI | 2020/5/28 | 19 | 2491 | No |  |

# Table S3. Age distribution of COVID-19 cases

| **Reference** | **Location** | **Study period** | **Study population** | **Data source** | **No. participants** | **Proportions of COVID-19 cases by age (years)**  **(%)** | | | |  | **Age-stratified incidence of COVID-19 cases**  **(per 100,000 population)** | | | |
| --- | --- | --- | --- | --- | --- | --- | --- | --- | --- | --- | --- | --- | --- | --- |
|  |  |  |  |  |  | **<20** | **20-39** | **40-59** | **60+** |  | **<20** | **20-39** | **40-59** | **60+** |
| Pan A (JAMA)[79] | Wuhan, China | 2019/12/8-2020/3/8 | All reported lab-confirmed COVID-19 cases | Notifiable Disease Report System | 32,583 | 1.6 | 18.3 | 37.7 | 42.4 |  | 4.0 | 24.0 | 51.6 | 84.4 |
| COVID-19 National Incident Room Surveillance Team of Australia (Commun Dis Intell)[80]* | Australia | 2020/1/13-2020/5/17 | All reported lab-confirmed COVID-19 cases | National Notifiable Diseases Surveillance System | 7,075 | 4.6 | 35.7 | 29.3 | 30.3 |  | 5.0 | 35.0 | 31.8 | 39.4 |
| Stokes EK (MWR Morb Mortal Wkly Rep)[81] | United States | 2020/1/22-2020/5/30 | All reported lab-confirmed COVID-19 cases | Reported to US CDC | 1,320,488 | 5.3 | 30.1 | 34.5 | 30.2 |  | 85.0 | 445.7 | 546.2 | 547.7 |
| Cruz CJP (PLoS One)[82] | Hong Kong, China | 2020/1/23-2020/4/16 | All reported lab-confirmed COVID-19 cases | Centre for Health Protection of the Hong Kong Department of Health | 1,017 | 14.6 | 45.8 | 24.6 | 15.0 |  | 12.7 | 24.8 | 10.9 | 9.2 |
| Gujski M (Med Sci Monit)[83] | Poland | 2020/3/3-  2020/3/27 | All reported lab-confirmed COVID-19 cases | Chief Sanitary Inspectorate | 1,157 | 7.5 | 30.2 | 37.6 | 24.9 |  | 1.2 | 3.2 | 4.2 | 3.0 |
| Mazumder A (F1000Research)[84]* | India | 2020/3/1-  2020/4/14 | Among 10,939 lab-confirmed COVID-19 cases, those with age information were included | State government and central government agencies | 1,161 | 9.4 | 43.2 | 31.0 | 16.5 |  | 0.0 | 0.1 | 0.1 | 0.1 |
| Jung C Y (Int J Infect Dis)[85]* | South Korea | 2020/1/24-  2020/4/9 | All reported lab-confirmed COVID-19 cases | Korea Centers for Disease Control and Prevention | 10237 | 4.7 | 38.5 | 31.4 | 25.4 |  | 5.2 | 28.4 | 19.5 | 21.9 |
| BULUT C (Turk J Med Sci)[86]* | Spain | 2020/1/31-  2020/4/6 | All reported lab-confirmed COVID-19 cases | Spanish Ministry of Health | 88,190 | 0.8 | 15.2 | 34.0 | 50.0 |  | 8.3 | 134.1 | 200.0 | 351.4 |
| National Health Service[87]* | United Kingdom | 2020/1/30-2020/8/4 | All reported lab-confirmed COVID-19 cases, those without age information were included | National Health Service | 260,695 | 4.0 | 25.2 | 29.9 | 41.0 |  | 66.7 | 378.3 | 441.4 | 640.6 |
| Robert Koch Institute[88]* | Germany | 2020/1/3-2020/8/6 | All reported lab-confirmed COVID-19 cases | Robert Koch Institute Information System | 212,594 | 8.4 | 29.6 | 33.5 | 28.5 |  | 115.5 | 311.5 | 301.5 | 253.0 |

* These studies reported the number of COVID-19 cases, but did not report age-specific incidence rates. We obtained the country-specific population size by age groups from UN mid-year population estimates for 2020 (<https://population.un.org/wpp/Download/Standard/Population/>) (accessed July 15, 2020). Age-stratified cumulative incidence was then calculated by dividing the number of COVID-19 cases over the number of populations in each age group. The reported number of COVID-19 cases and population size were shown in Table S5.

# Table S4. Data used to estimate the COVID-19 incidence rate

| **Reference** | **No. participants** | **Number of COVID-19 cases**  **by age group** | | | |  | **Population by age group**  **(thousand)*** | | | |
| --- | --- | --- | --- | --- | --- | --- | --- | --- | --- | --- |
|  |  | **<20** | **20-39** | **40-59** | **60+** |  | **<20** | **20-39** | **40-59** | **60+** |
| COVID-19 National Incident Room Surveillance Team of Australia (Commun Dis Intell) [80] | 7,075 | / | / | / | 2,144 |  | 6,439 | 7,094 | 6,414 | 5,553 |
| Mazumder A (F1000Research) [84] | 1,161 | 109 | 502 | 360 | 192 |  | 487,064 | 455,538 | 297,792 | 139,610 |
| Jung C Y (Int J Infect Dis) [85] | 10,237 | 481 | 3,947 | 3,206 | 2,603 |  | 9,331 | 13,878 | 16,434 | 11,864 |
| BULUT C (Turk J Med Sci) [86] | 88,190 | 741 | 13,390 | 29,998 | 44,061 |  | 8,924 | 9,989 | 15,023 | 12,524 |
| National Health Service[87] | 260,695 | 10,471 | 65,513 | 77,866 | 106,845 |  | 15,698 | 17,317 | 17,642 | 16,678 |
| Robert Koch Institute[88] | 212,594 | 17,914 | / | / | / |  | 15,812 | 20,249 | 23,732 | 23,991 |

*We obtained the country-specific population size by age groups from UN mid-year population estimates for 2020 (<https://population.un.org/wpp/Download/Standard/Population/>) (accessed July 15, 2020).

# Figure S2. Pooled incidence of COVID-19 cases, stratified by age.


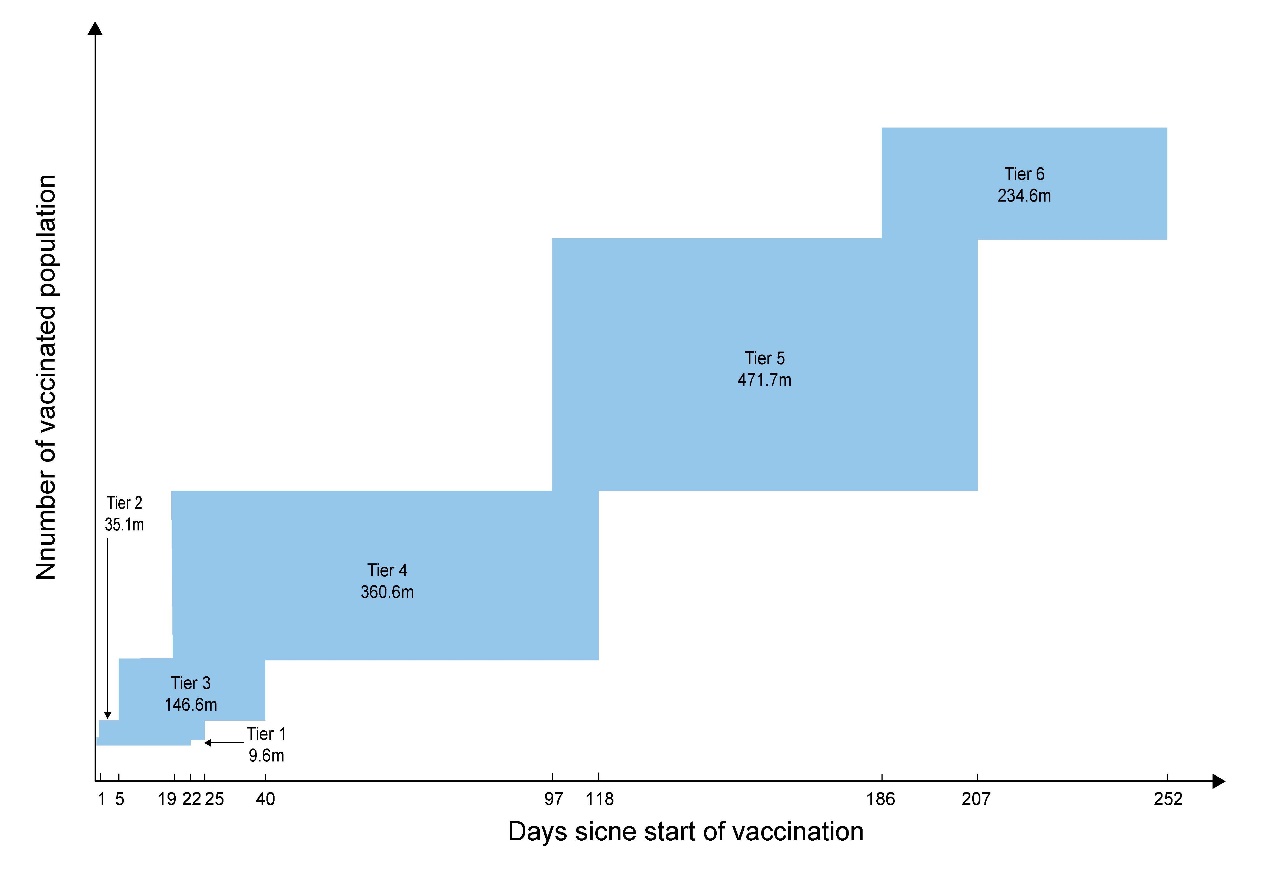


# Figure S3. Sensitivity analyses on days needed to vaccinate 90% of the target population, stratified by vaccination tier, under the assumption that 10 million doses are administered per day. Note that values reported within the square denote 90% of the population size in each tier; m denotes million.


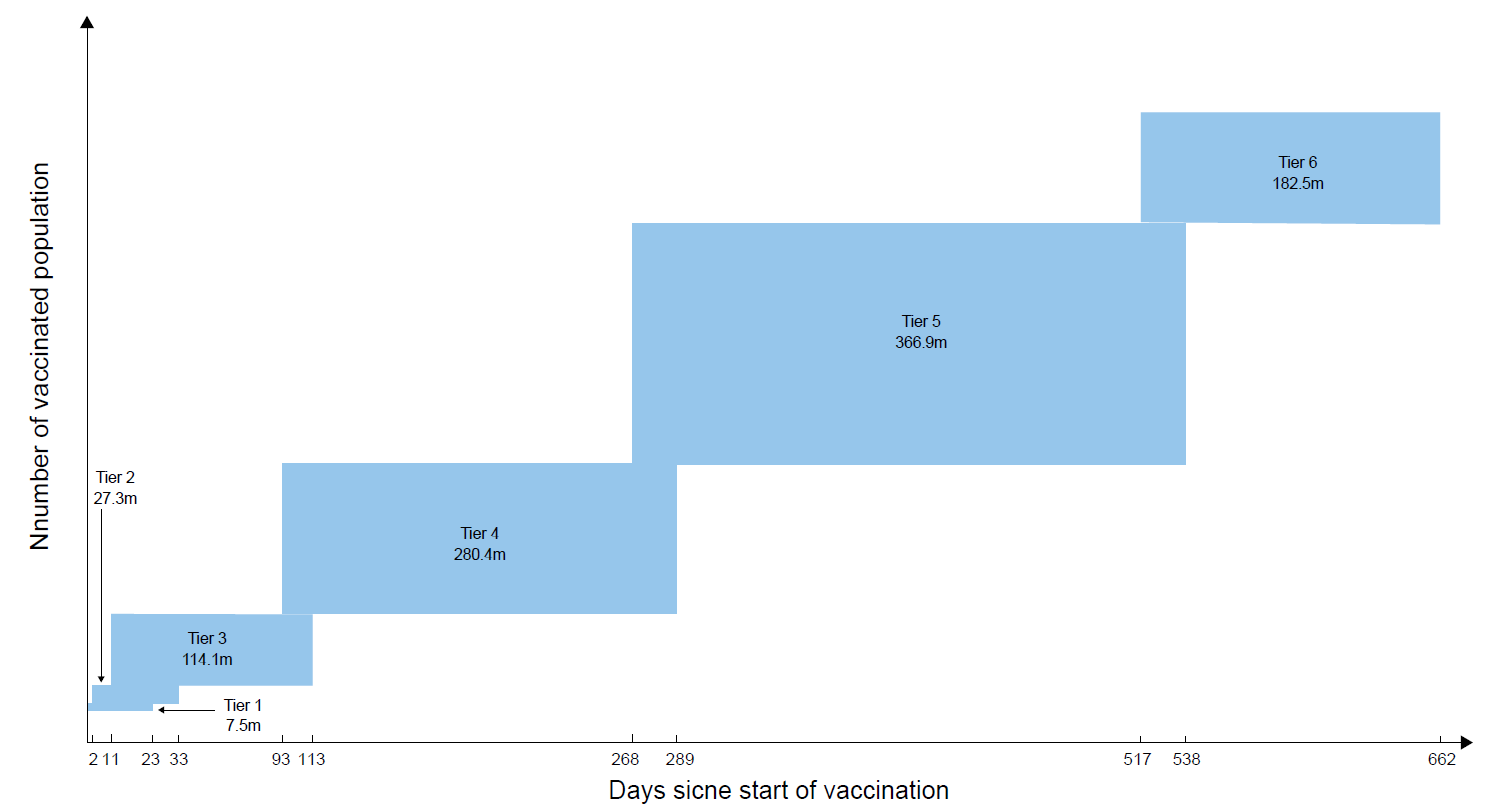


# Figure S4. Sensitivity analyses on days needed to vaccinate 70% of the target population, stratified by vaccination tier, under the assumption that 3 million doses are administered per day. Note that values reported within the square denote 70% of the population size in each tier; m denotes million.


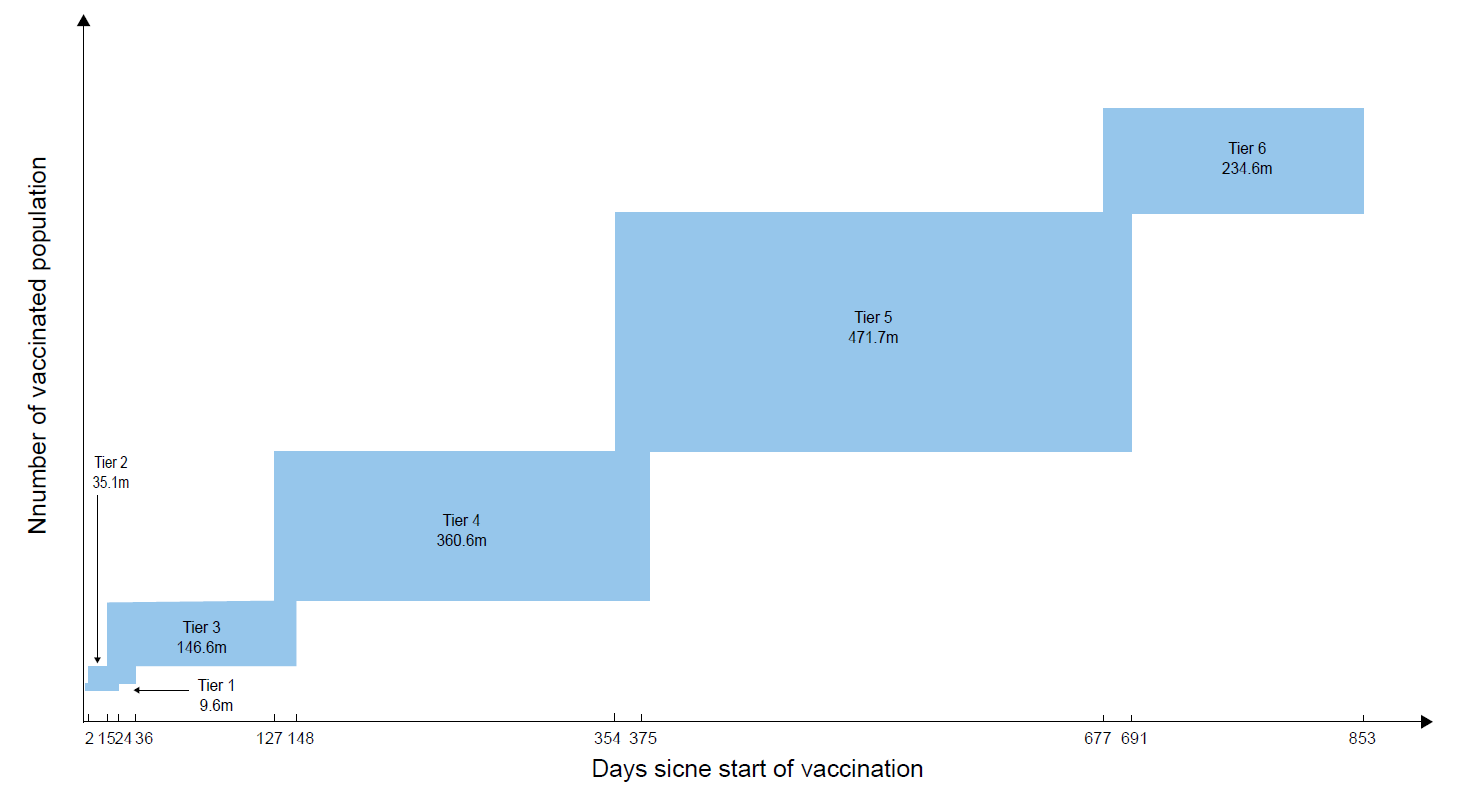


# Figure S5. Sensitivity analyses on days needed to vaccinate 90% of the target population, stratified by vaccination tier, under the assumption that 3 million doses are administered per day. Note that values reported within the square denote 90% of the population size in each tier; m denotes million.


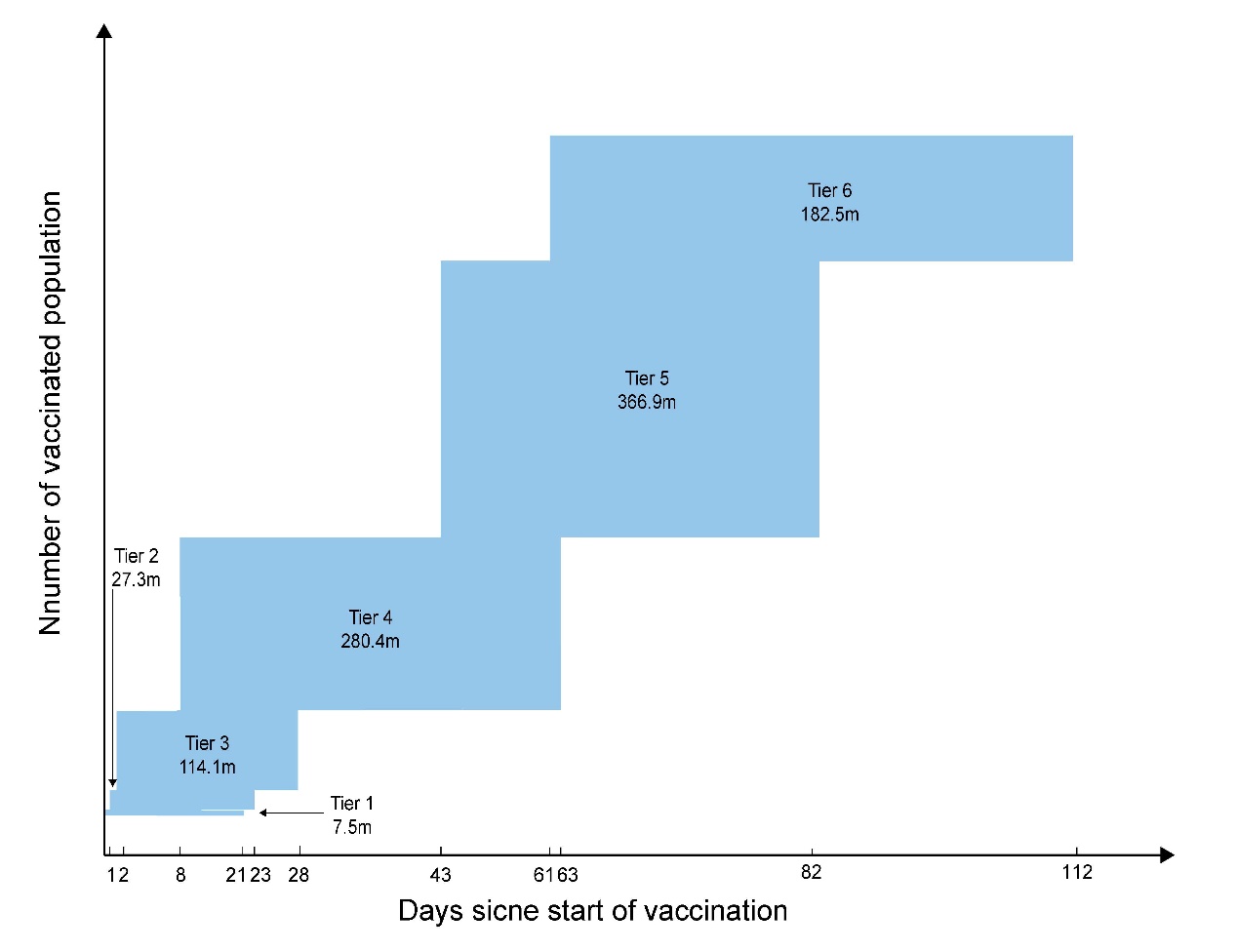


# Figure S6. Sensitivity analyses on days needed to vaccinate 70% of the target population, stratified by vaccination tier, under the assumption that 20 million doses are administered per day. Note that values reported within the square denote 70% of the population size in each tier; m denotes million.


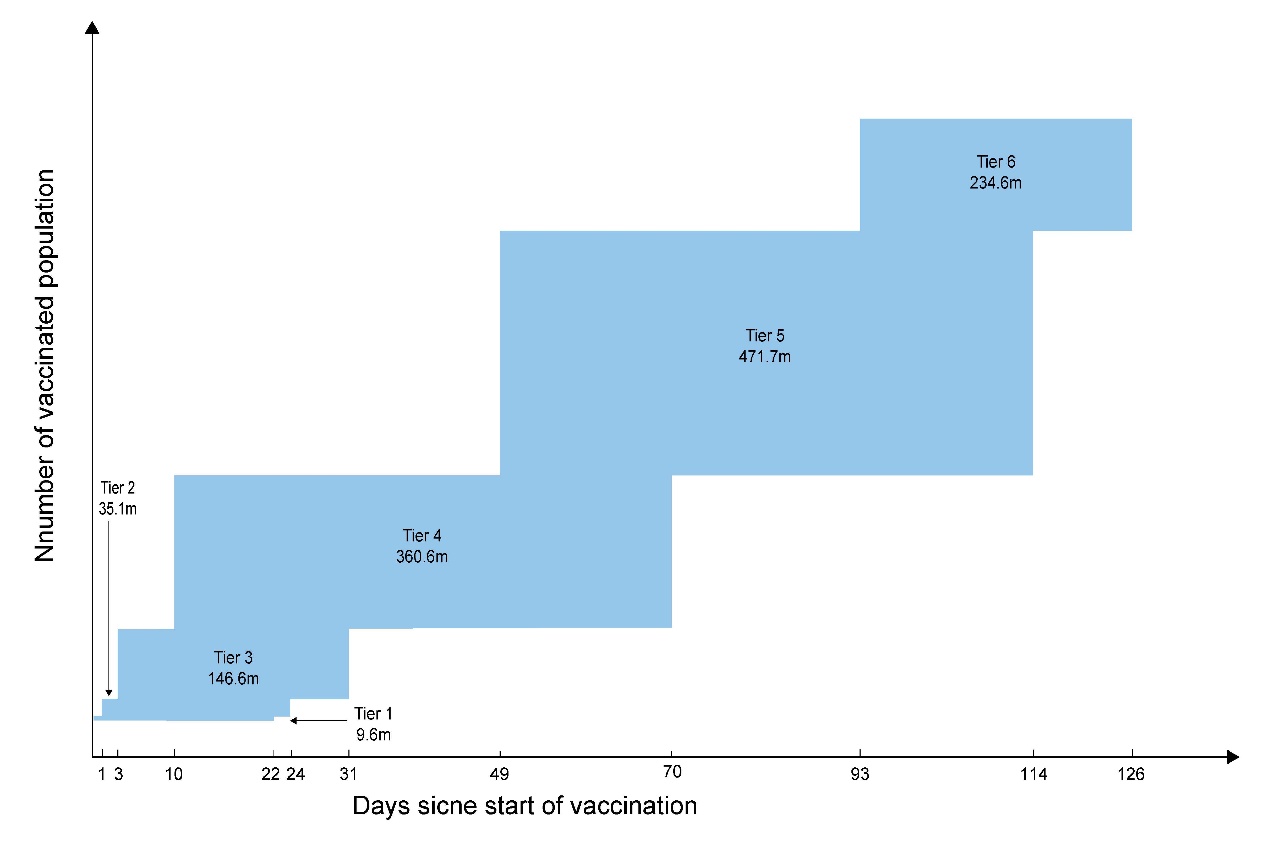


# Figure S7. Sensitivity analyses on days needed to vaccinate 90% of the target population, stratified by vaccination tier, under the assumption that 20 million doses are administered per day. Note that values reported within the square denote 90% of the population size in each tier; m denotes million.

# References

1. World Health Organization. WHO SAGE Values Framework for The Allocation and Prioritization of COVID-19 Vaccination. <https://apps.who.int/iris/bitstream/handle/10665/334299/WHO-2019-nCoV-SAGE_Framework-Allocation_and_prioritization-2020.1-eng.pdf?ua=1>. Accessed 22 Sept 2020.

2. National Academies of Sciences E, and Medicine. Framework for Equitable Allocation of COVID-19 Vaccine. 2020. <https://doi.org/10.17226/25917> Accessed 30 Nov 2020.

3. The Joint Committee on Vaccination and Immunisation. JCVI: updated interim advice on priority groups for COVID-19 vaccination. <https://www.gov.uk/government/publications/priority-groups-for-coronavirus-covid-19-vaccination-advice-from-the-jcvi-25-september-2020/jcvi-updated-interim-advice-on-priority-groups-for-covid-19-vaccination>. Accessed 30 Nov 2020.

4. Zheng Z, Peng F, Xu B, Zhao J, Liu H, Peng J, Li Q, Jiang C, Zhou Y, Liu S et al. Risk factors of critical & mortal COVID-19 cases: A systematic literature review and meta-analysis. J Infect. 2020;81:e16-e25.

5. Singh AK, Gillies CL, Singh R, Singh A, Chudasama Y, Coles B, Seidu S, Zaccardi F, Davies MJ, Khunti K. Prevalence of co-morbidities and their association with mortality in patients with COVID-19: A systematic review and meta-analysis. Diabetes Obes Metab. 2020;22(10):1915-24.

6. Wang X, Fang X, Cai Z, Wu X, Gao X, Min J, Wang F. Comorbid Chronic Diseases and Acute Organ Injuries Are Strongly Correlated with Disease Severity and Mortality among COVID-19 Patients: A Systemic Review and Meta-Analysis. Research. 2020:2402961.

7. Taylor E, Hofmeyr R, Torborg A, Tonder CV, Anaesthesia BBJSAJo, Analgesia. Risk factors and interventions associated with mortality or survival in adult COVID-19 patients admitted to critical care: a systematic review and meta-analysis. South Afr J Anaesth Analg. 2020;26(3):116-27.

8. Wang B, Li R, Lu Z, Huang Y. Does comorbidity increase the risk of patients with COVID-19: evidence from meta-analysis. Aging. 2020;12(7):6049-57.

9. Jain V, Yuan JM. Predictive symptoms and comorbidities for severe COVID-19 and intensive care unit admission: a systematic review and meta-analysis. Int J Public Health. 2020;65(5):533-46.

10. Giannakoulis VG, Papoutsi E, Siempos I. Effect of Cancer on Clinical Outcomes of Patients With COVID-19: A Meta-Analysis of Patient Data. JCO Glob Oncol. 2020;6:799-808.

11. Tian W, Jiang W, Yao J, Nicholson CJ, Li RH, Sigurslid HH, Wooster L, Rotter JI, Guo X, Malhotra R. Predictors of mortality in hospitalized COVID-19 patients: A systematic review and meta-analysis. J Med Virol. 2020;<https://doi.org/10.1002/jmv.26050>.

12. Li J, He X, Yuan Y, Zhang W, Li X, Zhang Y, Li S, Guan C, Gao Z, Dong G. Meta-analysis investigating the relationship between clinical features, outcomes, and severity of severe acute respiratory syndrome coronavirus 2 (SARS-CoV-2) pneumonia. Am J Infect Control. 2020;<https://doi.org/10.1016/j.ajic.2020.06.008>.

13. Wu ZH, Tang Y, Cheng Q. Diabetes increases the mortality of patients with COVID-19: a meta-analysis. Acta Diabetol. 2020:1-6.

14. Xu L, Mao Y, Chen G. Risk factors for 2019 novel coronavirus disease (COVID-19) patients progressing to critical illness: a systematic review and meta-analysis. Aging (Albany NY). 2020;12(12):12410-21.

15. Ssentongo P, Ssentongo AE, Heilbrunn ES, Ba DM, Chinchilli VM. Association of cardiovascular disease and 10 other pre-existing comorbidities with COVID-19 mortality: A systematic review and meta-analysis. PloS one. 2020;15(8):e0238215.

16. Bellou V, Tzoulaki I, Evangelou E, Belbasis L. Risk factors for adverse clinical outcomes in patients with COVID-19: A systematic review and meta-analysis. medRxiv. 2020;<https://doi.org/10.1101/2020.05.13.20100495>.

17. Rahman A, Sathi NJ. Risk Factors of the Severity of COVID-19: a Meta-Analysis. Journal. 2020;<https://www.medrxiv.org/content/medrxiv/early/2020/05/10/2020.04.30.20086744.full.pdf>.

18. Khan MMA, Khan MN, Mustagir MG, Rana J, Islam MS, Kabir MI. Effects of underlying morbidities on the occurrence of deaths in COVID-19 patients: A systematic review and meta-analysis. J Glob Health. 2020;10(2):020503.

19. Soeroto AY, Soetedjo NN, Purwiga A, Santoso P, Kulsum ID, Suryadinata H, Ferdian F. Effect of increased BMI and obesity on the outcome of COVID-19 adult patients: A systematic review and meta-analysis. Diabetes Metab Syndr. 2020;14(6):1897-904.

20. Liu Y, Zhang Z, Pan X, Xing G, Zhang Y, Liu Z, Tu S. The Chronic Kidney Disease and Acute Kidney Injury Involvement in COVID-19 Pandemic: A Systematic Review and Meta-analysis. medRxiv. 2020;<https://doi.org/10.1101/2020.05.08.20095968>.

21. Matsushita K, Ding N, Kou M, Hu X, Chen M, Gao Y, Honda Y, Zhao D, Dowdy D, Mok Y et al. The Relationship of COVID-19 Severity with Cardiovascular Disease and Its Traditional Risk Factors: A Systematic Review and Meta-Analysis. Glob Heart. 2020;15(1):64.

22. Tabrizi R, Lankarani KB, Nowrouzi-sohrabi P, Shabani-Borujeni M, Rezaei S, Hosseini-bensenjan M, vakili S, Heydari ST, Ashraf MA. The role of comorbidities and clinical predictors of severe disease in COVID-19: a systematic review and meta-analysis. medRxiv. 2020;<https://doi.org/10.1101/2020.04.21.20074633>.

23. Dorjee K, Kim H. Epidemiological Risk Factors Associated with Death and Severe Disease in Patients Suffering From COVID-19: A Comprehensive Systematic Review and Meta-analysis. medRxiv. 2020;<https://doi.org/10.1101/2020.06.19.20135483>.

24. Hessami A, Shamshirian A, Heydari K, Pourali F, Alizadeh-Navaei R, Moosazadeh M, Abrotan S, Shojaei L, Sedighi S, Shamshirian D et al. Cardiovascular Diseases and COVID-19 Mortality and Intensive Care Unit Admission: A Systematic Review and Meta-analysis. medRxiv. 2020;<https://doi.org/10.1101/2020.04.12.20062869>.

25. Chen Y, Gong X, Wang L, Guo J. Effects of hypertension, diabetes and coronary heart disease on COVID-19 diseases severity: a systematic review and meta-analysis. medRxiv. 2020;<https://doi.org/10.1101/2020.03.25.20043133>.

26. Parohan M, Yaghoubi S, Seraji A, Javanbakht MH, Sarraf P, Djalali M. Risk factors for mortality in patients with Coronavirus disease 2019 (COVID-19) infection: a systematic review and meta-analysis of observational studies. Aging Male. 2020:1-9.

27. Zhao X, Zhang B, Li P, Ma C, Gu J, Hou P, Guo Z, Wu H, Bai Y. Incidence, clinical characteristics and prognostic factor of patients with COVID-19: a systematic review and meta-analysis. medRxiv. 2020;<https://doi.org/10.1101/2020.03.17.20037572>.

28. Palaiodimos L, Chamorro-Pareja N, Karamanis D, Li W, Zavras PD, Chang KM, Mathias P, Kokkinidis DG. Diabetes is associated with increased risk for in-hospital mortality in patients with COVID-19: a systematic review and meta-analysis comprising 18,506 patients. Hormones (Athens). 2020:1-10.

29. Jutzeler CR, Bourguignon L, Weis CV, Tong B, Wong C, Rieck B, Pargger H, Tschudin-Sutter S, Egli A, Borgwardt K et al. Comorbidities, clinical signs and symptoms, laboratory findings, imaging features, treatment strategies, and outcomes in adult and pediatric patients with COVID-19: A systematic review and meta-analysis. Travel Med Infect Dis. 2020:101825.

30. Islam MS, Barek MA, Aziz MA, Aka TD, Jakaria M. Association of age, sex, comorbidities, and clinical symptoms with the severity and mortality of COVID-19 cases: a meta-analysis with 85 studies and 67299 cases. medRxiv. 2020;<https://doi.org/10.1101/2020.05.23.20110965>.

31. Zaki N, Mohamed EA, Ibrahim S, Khan G. The influence of comorbidity on the severity of COVID-19 disease: systematic review and analysis. medRxiv. 2020;<https://doi.org/10.1101/2020.06.18.20134478>.

32. Cao G, Li P, Cheng Y, Chen B, Wang S, Wang Z, Xiong M, Zheng R, Guo M, Sun Q. A risk prediction model for evaluating the disease progression of covid-19 pneumonia based on meta-analysis and 214 clinical cases. SSRN. 2020;<http://doi.org/10.2139/ssrn.3569869>.

33. Salunke AA, Nandy K, Pathak SK, Shah J, Kamani M, Kottakota V, Thivari P, Pandey A, Patel K, Rathod P et al. Impact of COVID -19 in cancer patients on severity of disease and fatal outcomes: A systematic review and meta-analysis. Diabetes Metab Syndr. 2020;14(5):1431-7.

34. Sreenivasan J, Khan MS, Anker SD, Kaul R, Khan SU, Metra M, Senni M, Cooper HA, Zhang Y, Zhang J et al. Cardiovascular Risk Factors and Complications in Patients Infected with COVID-19_ A Systematic Review. SSRN. 2020;<http://doi.org/10.2139/ssrn.3569855>.

35. Biswas M, Rahaman S, Biswas TK, Haque Z, Ibrahim B. Effects of Sex, Age and Comorbidities on the Risk of Infection and Death Associated with COVID-19_ A Meta-Analysis of 47807 Confirmed Cases. SSRN. 2020;<http://doi.org/10.2139/ssrn.3566146>.

36. Kumar A, Arora A, Sharma P, Anikhindi SA, Bansal N, Singla V, Khare S, Srivastava A. Clinical Features of COVID-19 and Factors Associated with Severe Clinical Course: A Systematic Review and Meta-analysis. SSRN. 2020;<http://doi.org/10.2139/ssrn.3566166>.

37. Aggarwal G, Cheruiyot I, Aggarwal S, Wong J, Lippi G, Lavie CJ, Henry BM, Sanchis-Gomar F. Association of Cardiovascular Disease With Coronavirus Disease 2019 (COVID-19) Severity: A Meta-Analysis. Curr Probl Cardiol. 2020;45(8):100617.

38. Tian Y, Qiu X, Wang C, Zhao J, Jiang X, Niu W, Huang J, Zhang F. Cancer associates with risk and severe events of COVID-19: A systematic review and meta-analysis. Int J Cancer. 2020;<https://doi.org/10.1002/ijc.33213>.

39. Guo L, Shi Z, Zhang Y, Wang C, Do Vale Moreira NC, Zuo H, Hussain A. Comorbid diabetes and the risk of disease severity or death among 8807 COVID-19 patients in China: A meta-analysis. Diabetes Res Clin Pract. 2020;166:108346.

40. Zhou Y, Yang Q, Chi J, Dong B, Lv W, Shen L, Wang Y. Comorbidities and the risk of severe or fatal outcomes associated with coronavirus disease 2019: A systematic review and meta-analysis. Int J Infect Dis. 2020;99:47-56.

41. Mantovani A, Byrne CD, Zheng MH, Targher G. Diabetes as a risk factor for greater COVID-19 severity and in-hospital death: A meta-analysis of observational studies. Nutr Metab Cardiovasc Dis. 2020;30(8):1236-48.

42. Fang X, Li S, Yu H, Wang P, Zhang Y, Chen Z, Li Y, Cheng L, Li W, Jia H et al. Epidemiological, comorbidity factors with severity and prognosis of COVID-19: a systematic review and meta-analysis. Aging. 2020;12(13):12493-503.

43. Li X, Guan B, Su T, Liu W, Chen M, Bin Waleed K, Guan X, Gary T, Zhu Z. Impact of cardiovascular disease and cardiac injury on in-hospital mortality in patients with COVID-19: a systematic review and meta-analysis. Heart. 2020;106(15):1142-7.

44. Pranata R, Huang I, Lim MA, Wahjoepramono EJ, July J. Impact of cerebrovascular and cardiovascular diseases on mortality and severity of COVID-19-systematic review, meta-analysis, and meta-regression. J Stroke Cerebrovasc. 2020;29(8):104949.

45. Hussain A, Mahawar K, Xia Z, Yang W, El-Hasani S. Obesity and mortality of COVID-19. Meta-analysis. Obes Res Clin Pract. 2020;14:295-300.

46. Földi M, Farkas N, Kiss S, Zádori N, Váncsa S, Szakó L, Dembrovszky F, Solymár M, Bartalis E, Szakács Z et al. Obesity is a risk factor for developing critical condition in COVID-19 patients: A systematic review and meta-analysis. Obes Rev. 2020;21:e13095.

47. Figliozzi S, Masci PG, Ahmadi N, Tondi L, Koutli E, Aimo A, Stamatelopoulos K, Dimopoulos MA, Lp Caforio A, Georgiopoulos G. Predictors of Adverse Prognosis in Covid-19: A Systematic Review and Meta-analysis. Eur J Clin Invest. 2020:e13362.

48. Kovalic AJ, Satapathy SK, Thuluvath PJ. Prevalence of chronic liver disease in patients with COVID-19 and their clinical outcomes: a systematic review and meta-analysis. Hepatol Int. 2020;14(5):612-20.

49. Nandy K, Salunke A, Pathak SK, Pandey A, Doctor C, Puj K, Sharma M, Jain A, Warikoo V. Coronavirus disease (COVID-19): A systematic review and meta-analysis to evaluate the impact of various comorbidities on serious events. Diabetes Metab Syndr. 2020;14(5):1017-25.

50. Zhang J, Wu J, Sun X, Xue H, Shao J, Cai W, Jing Y, Yue M, Dong C. Association of hypertension with the severity and fatality of SARS-CoV-2 infection: A meta-analysis. Epidemiol Infect. 2020;148:e106.

51. Huang I, Lim MA, Pranata R. Diabetes mellitus is associated with increased mortality and severity of disease in COVID-19 pneumonia - A systematic review, meta-analysis, and meta-regression. Diabetes Metab Syndr. 2020;14(4):395-403.

52. Parveen R, Sehar N, Bajpai R, Agarwal NB. Association of diabetes and hypertension with disease severity in covid-19 patients: A systematic literature review and exploratory meta-analysis. Diabetes Res Clin Pract. 2020;166:108295.

53. Ofori-Asenso R, Ogundipe O, Agyeman AA, Chin KL, Mazidi M, Ademi Z, De Bruin ML, Liew D. Cancer is associated with severe disease in COVID-19 patients: a systematic review and meta-analysis. Ecancermedicalscience. 2020;14:1047.

54. Lu L, Zhong W, Bian Z, Li Z, Zhang K, Liang B, Zhong Y, Hu M, Lin L, Liu J et al. A comparison of mortality-related risk factors of COVID-19, SARS, and MERS: A systematic review and meta-analysis. J Infect. 2020;81:e18-e25.

55. Liu M, Gao Y, Zhang Y, Shi S, Chen Y, Tian J. The association between severe or dead COVID-19 and autoimmune diseases: A systematic review and meta-analysis. J Infect. 2020;81:e93-e5.

56. Liu H, Chen S, Liu M, Nie H, Lu H. Comorbid Chronic Diseases are Strongly Correlated with Disease Severity among COVID-19 Patients: A Systematic Review and Meta-Analysis. Aging Dis. 2020;11(3):668-78.

57. Lippi G, Wong J, Henry BM. Hypertension in patients with coronavirus disease 2019 (COVID-19): a pooled analysis. Pol Arch Intern Med. 2020;130(4):304-9.

58. Aggarwal G, Lippi G, Lavie CJ, Henry BM, Sanchis-Gomar F. Diabetes Mellitus Association with Coronavirus Disease 2019 (COVID-19) Severity and Mortality: A Pooled Analysis. Journal of diabetes. 2020;12(11):851-5.

59. Zhao Q, Meng M, Kumar R, Wu Y, Huang J, Lian N, Deng Y, Lin S. The impact of COPD and smoking history on the severity of COVID-19: A systemic review and meta-analysis. J Med Virol. 2020;<https://doi.org/10.1002/jmv.25889>.

60. Zhao J, Li X, Gao Y, Huang W. Risk factors for the exacerbation of patients with 2019 Novel Coronavirus: A meta-analysis. Int J Med Sci. 2020;17(12):1744-50.

61. Yang J, Zheng Y, Gou X, Pu K, Chen Z, Guo Q, Ji R, Wang H, Wang Y, Zhou Y. Prevalence of comorbidities and its effects in patients infected with SARS-CoV-2: a systematic review and meta-analysis. Int J Infect Dis. 2020;94:91-5.

62. Yang J, Hu J, Zhu C. Obesity aggravates COVID-19: a systematic review and meta-analysis. J Med Virol. 2020;<https://doi.org/10.1002/jmv.26237>.

63. Wu J, Zhang J, Sun X, Wang L, Xu Y, Zhang Y, Liu X, Dong C. Influence of diabetes mellitus on the severity and fatality of SARS-CoV-2 (COVID-19) infection. Diabetes Obes Metab. 2020;22(10):1907-14.

64. Pranata R, Lim MA, Yonas E, Vania R, Lukito AA, Siswanto BB, Meyer M. Body Mass Index and Outcome in Patients with COVID-19: A Dose-Response Meta-Analysis. Diabetes Metab. 2020;S1262-3636(20):30097-5.

65. Kumar A, Arora A, Sharma P, Anikhindi SA, Bansal N, Singla V, Khare S, Srivastava A. Is diabetes mellitus associated with mortality and severity of COVID-19? A meta-analysis. Diabetes Metab Syndr. 2020;14(4):535-45.

66. Alqahtani JS, Oyelade T, Aldhahir AM, Alghamdi SM, Almehmadi M, Alqahtani AS, Quaderi S, Mandal S, Hurst JR. Prevalence, Severity and Mortality associated with COPD and Smoking in patients with COVID-19: A Rapid Systematic Review and Meta-Analysis. PloS one. 2020;15(5):e0233147.

67. Liu M, Gao Y, Shi S, Chen Y, Yang K, Tian J. Drinking no-links to the severity of COVID-19: a systematic review and meta-analysis. J Infect. 2020;81(2):e126-e7.

68. Gao Y, Chen Y, Liu M, Shi S, Tian J. Impacts of immunosuppression and immunodeficiency on COVID-19: A systematic review and meta-analysis. J Infect. 2020;81(2):e93-e5.

69. Gao Y, Liu M, Chen Y, Shi S, Geng J, Tian J. Association between tuberculosis and COVID-19 severity and mortality: A rapid systematic review and meta-analysis. J Med Virol. 2020;<https://doi.org/10.1002/jmv.26311>.

70. ElGohary GM, Hashmi S, Styczynski J, Kharfan-Dabaja MA, Alblooshi RM, de la Cámara R, Mohmed S, Alshaibani A, Cesaro S, Abd El-Aziz N et al. The risk and prognosis of COVID-19 infection in cancer patients: A systematic review and meta-analysis. Hematol Oncol Stem Cell Ther. 2020;S1658-3876(20):30122-9.

71. Wu X, Liu L, Jiao J, Yang L, Zhu B, Li X. Characterisation of clinical, laboratory and imaging factors related to mild vs. severe covid-19 infection: a systematic review and meta-analysis. Ann Med. 2020;52(7):334-44.

72. Siepmann T, Sedghi A, Barlinn J, de With K, Mirow L, Wolz M, Gruenewald T, Helbig S, Schroettner P, Winzer S et al. Association of history of cerebrovascular disease with severity of COVID-19. J Neurol. 2020:1-12.

73. Sanchez-Ramirez DC, Mackey D. Underlying respiratory diseases, specifically COPD, and smoking are associated with severe COVID-19 outcomes: A systematic review and meta-analysis. Respir Med. 2020;171:106096.

74. Deng M, Ye M, Xiao X, Liu J, Xia Z, Luo X, Jiang L, Kang S, Zuo Z, Wu T. Multi-organ Dysfunction in Patients with COVID-19: A Systematic Review and Meta-analysis. Aging and disease. 2020;11(4):874-94.

75. Patel U, Malik P, Shah D, Patel A, Dhamoon M, Jani V. Pre-existing cerebrovascular disease and poor outcomes of COVID-19 hospitalized patients: a meta-analysis. J Neurol. 2020:1-8.

76. Sales-Peres SHC, de Azevedo-Silva LJ, Bonato RCS, Sales-Peres MC, Pinto A, Santiago Junior JF. Coronavirus (SARS-CoV-2) and the risk of obesity for critically illness and ICU admitted: Meta-analysis of the epidemiological evidence. Obes Res Clin Pract. 2020.

77. Hariyanto TI, Kurniawan A. Dyslipidemia is associated with severe coronavirus disease 2019 (COVID-19) infection. Diabetes Metab Syndr. 2020;14(5):1463-5.

78. Yin T, Li Y, Ying Y, Luo Z. Association of comorbidity with COVID-19 in Chinese population Analysis of risk factors of the COVID-19 severity. SSRN. 2020;<http://dx.doi.org/10.2139/ssrn.3624220>.

79. Pan A, Liu L, Wang C, Guo H, Hao X, Wang Q, Huang J, He N, Yu H, Lin X et al. Association of Public Health Interventions With the Epidemiology of the COVID-19 Outbreak in Wuhan, China. JAMA. 2020;323(19):1915-23.

80. COVID-19, Australia: Epidemiology Report 16 (Reporting week to 23:59 AEST 17 May 2020). Commun Dis Intell (2018). 2020;44.

81. Stokes EK, Zambrano LD, Anderson KN, Marder EP, Raz KM, El Burai Felix S, Tie Y, Fullerton KE. Coronavirus Disease 2019 Case Surveillance - United States, January 22-May 30, 2020. MMWR Morb Mortal Wkly Rep. 2020;69(24):759-65.

82. Cruz CJP, Ganly R, Li Z, Gietel-Basten S. Exploring the young demographic profile of COVID-19 cases in Hong Kong: Evidence from migration and travel history data. PloS one. 2020;15(6):e0235306.

83. Gujski M, Raciborski F, Jankowski M, Nowicka PM, Rakocy K, Pinkas J. Epidemiological Analysis of the First 1389 Cases of COVID-19 in Poland: A Preliminary Report. Med Sci Monit. 2020;26:e924702.

84. Mazumder A, Arora M, Bharadiya V, Berry P, Agarwal M, Behera P, Shewade HD, Lohiya A, Gupta M, Rao A et al. SARS-CoV-2 epidemic in India: epidemiological features and in silico analysis of the effect of interventions. F1000Res. 2020;9:315.

85. Jung CY, Park H, Kim DW, Choi YJ, Kim SW, Chang TI. Clinical Characteristics of Asymptomatic Patients with COVID-19: A Nationwide Cohort Study in South Korea. Int J Infect Dis. 2020;99:266-8.

86. Bulut C, Kato Y. Epidemiology of COVID-19. Turk J Med Sci. 2020;50(SI-1):563-70.

87. Public Health England. The weekly surveillance report in England. <https://assets.publishing.service.gov.uk/government/uploads/system/uploads/attachment_data/file/907429/COVID19_Weekly_Report_04_August_New_caveat.pdf>. Accessed 7 Aug 2020.

88. Robert Koch Institute. Coronavirus Disease 2019 (COVID-19) Daily Situation Report of the Robert Koch Institute. <https://www.rki.de/DE/Content/InfAZ/N/Neuartiges_Coronavirus/Situationsberichte/2020-08-06-en.pdf?__blob=publicationFile>. Accessed 6 Aug 2020.
